# Periodontal and Peri-Implant Status Among Users of Different Inhaled Tobacco and Nicotine Products: A Systematic Review

**DOI:** 10.3390/dj14090562

**Published:** 2026-09-03

**Authors:** Maria Pia Di Palo, Federica Di Spirito, Francesco Giordano, Giuseppina De Benedetto, Roberto Lo Giudice, Federica Piedepalumbo, Gabriele Balsamo, Colomba Pessolano, Marina Garofano, Andrea Marino, Alessia Bramanti

**Affiliations:** 1 Department of Medicine, Surgery and Dentistry, University of Salerno, 84081 Baronissi, Italy; frgiordano@unisa.it (F.G.); giusydb15@gmail.com (G.D.B.); f.piedepalumbo1@studenti.unisa.it (F.P.); g.balsamo4@studenti.unisa.it (G.B.); cpessolano@unisa.it (C.P.); mgarofano@unisa.it (M.G.); andremarino@unisa.it (A.M.); abramanti@unisa.it (A.B.); 2 Department of Innovative Technologies in Medicine & Dentistry, University “G. d’Annunzio” Chieti-Pescara, 66100 Chieti, Italy; 3 Department of Human Pathology in Adulthood and Childhood “G. Barresi”, University Hospital “G. Martino” of Messina, Via Consolare Valeria 1, 98123 Messina, Italy; roberto.logiudice@unime.it

**Keywords:** periodontal health, periodontitis, peri-implant health, peri-implantitis, smokers, tobacco, cigarette smoking, waterpipe, heat-not-burn tobacco, electronic cigarette

## Abstract

**Background/Objectives**: Periodontal and peri-implant diseases are influenced by several risk factors, including smoking. While cigarette smoking (CS) is a well-established risk factor, the impact of heated tobacco products (HTPs), electronic cigarettes (E-Cigs), and waterpipe (WS) remains less clear. This systematic review aimed to update the current evidence evaluating the clinical, radiographical, and inflammatory biomarkers of periodontal and peri-implant status among different untreated adult users of inhaled tobacco and nicotine products. **Methods**: A systematic search of PubMed/MEDLINE, Scopus, and Cochrane Library was performed for studies published between April 2022 and December 2025. Data were extracted and synthesized qualitatively according to the study protocol (PROSPERO: CRD420261284851). Studies were qualitatively assessed using the Risk-of-Bias in Nonrandomized-I. **Results**: Thirty-one studies including 3106 subjects were analyzed. CS consistently showed the worst periodontal and peri-implant status, with higher plaque indices, probing depth (PD), clinical attachment level (CAL), marginal bone loss (MBL) and more missing teeth. WS showed parameters comparable to CS and worse than non-smokers, with particularly high plaque accumulation and probing depth, as well as a pro-inflammatory biomarker profile. E-Cigs and HTPs generally exhibited intermediate status; E-Cigs showed increased PD and CAL compared with non-smokers, with elevated bleeding on probing despite a lower gingival index, whereas MBL was notably higher, showing the highest overall values among all smoking categories, exceeding CS. HTPs presented slightly less harmful outcomes than other smokers, but the evidence was limited. Biomarker analysis indicated elevated pro-inflammatory and tissue-destructive markers, particularly in CS and WS. **Conclusions**: Smoking negatively affects periodontal and peri-implant status, with CS exhibiting the worst impact. E-Cigs and HTPs appear less harmful but are not risk-free. Longitudinal studies are warranted to define long-term effects, particularly on peri-implant status, for which robust evidence was restricted to CS.

## 1. Introduction

Periodontal diseases are microbial-mediated inflammatory processes around natural teeth associated with the host’s immunological response [1,2]. Gingivitis is the reversible manifestation of periodontal disease, which may progress to the irreversible form of periodontitis, which is characterized by the apical migration of the periodontal ligament, junctional epithelium, and bone levels, eventually leading to tooth loss [1,2].

Analogously, peri-implant diseases are inflammatory processes around dental implants, which are distinguished into peri-implant mucositis, the reversible manifestation mainly clinically characterized by bleeding on gentle probing, and peri-implantitis, the irreversible manifestation distinguished by the progressive loss of the supporting bone, eventually leading to implant failure [3].

Risk factors such as genetics, cigarette smoking, and diabetes mellitus have been associated with the disease susceptibility, prevalence, and severity [1].

In particular, cigarette smoking was identified as a grade modifier of periodontitis in a dose-dependent manner. In relation to smoking, non-smokers were associated with a slow rate of disease progression, smokers of <10 cigarettes per day with a moderate rate, and smokers of ≥10 cigarettes/day with a rapid rate [4].

Although cigarette smoking has been strongly associated with periodontitis, the majority of studies failed to determine cigarette smoking as a relevant predictor for peri-implantitis when adjustments for confounding and multivariate analyses were performed [5]. Currently, evidence about smoking as a predictor of peri-implantitis is inconclusive [5].

Following the introduction in the 1990s of flavored tobacco, the combination of water-cooled and flavored smoke has contributed to the constant increase in waterpipe tobacco smokers due to the distorted perception of decreased toxicity compared to traditional cigarettes [6,7]. Nonetheless, during a single session of waterpipe smoking of 30–60 min, waterpipe tobacco smokers inhale more than 40 L of smoke compared with about 1 L of a traditional cigarette [6]. As a consequence, available evidence suggests that waterpipe tobacco smoking results in significant exposure to a wide range of toxicants for users and may also affect non-users through environmental exposure, thereby representing a potential risk for adverse health outcomes [6]. Although the number of studies included in the latest systematic reviews published in 2015 and 2017, which evaluated the relationship between waterpipe and periodontitis, was limited, a significant association was registered (odds ratio ranging between 3.00 and 5.00) [7,8]. Furthermore, the other two systematic reviews focusing on peri-implant health reported statistically significantly worse peri-implant status among waterpipe smokers compared with non-smokers [9,10].

In contrast, current evidence regarding the impact of heated tobacco products (HTP) on periodontal and peri-implant health is less consistent, even if HTP is not relatively recent. HTP was initially developed in the late 1980s as an alternative to conventional cigarettes, with early devices such as Premier™ (R.J. Reynolds, 1988) (R.J. Reynolds Tobacco Company, Winston-Salem, NC, USA), followed by Eclipse™ (R.J. Reynolds Tobacco Company, Winston-Salem, NC, USA) and Accord™ (Philip Morris, Stamford, CT, USA) in the 1990s [11]. However, these early systems achieved limited market success and were subsequently withdrawn. The modern diffusion of HTP products began in 2014 with the introduction in Japan, Italy, and Switzerland of IQOS^®^ by Philip Morris International (Philip Morris International, Stamford, CT, USA), which led to their rapid global expansion [11].

Despite their increasing use, only a systematic review in 2022 [12] aimed at comparing conventional cigarette smokers, electronic cigarette users, and HTP users, but it failed to identify eligible clinical studies specifically addressing HTP exposure, thus preventing conclusions regarding their impact on periodontal and peri-implant tissues.

Nonetheless, emerging microbiological evidence by Mišković et al. (2024) [13] suggests that HTP use may influence the subgingival microbial ecosystem. When periodontal status and tobacco exposure are jointly considered, users of HTP appear to harbor a greater number of periopathogenic bacterial species compared to non-smokers, with microbial profiles approaching those observed in traditional cigarette smokers, particularly in the presence of periodontitis [13]. These findings support the hypothesis that HTP products may contribute to dysbiosis within periodontal and peri-implant niches, thereby reinforcing the need to comprehensively clarify their potential role in periodontal and peri-implant disease onset and progression.

Electronic cigarette smokers are a relatively new type of nicotine users [1]. Previous systematic reviews published in 2020 and 2022, which aimed to assess the impact of vaping on periodontal and peri-implant health, showed that the impact of vaping was relatively non-significant from a clinical point of view [1], and electronic cigarettes seem to be associated with attenuated clinical inflammatory signs of periodontitis and peri-implantitis compared with traditional cigarette smokers [12]. However, in consideration of the relatively recent introduction of vaping and that, at the biochemical level, electronic cigarette smokers exhibit increased pro-inflammatory cytokines (e.g., IL-1β, IL-6, TNF-α), oxidative stress markers, matrix metalloproteinases (MMPs), and altered RANKL/OPG balance compared to non-smokers, electronic cigarette users appear to exhibit a biologically active inflammatory and bone-resorptive environment [12]. The authors hypothesized that the investigation period was too short to capture all its effects, suggesting continuing to investigate how vaping influences the periodontal and peri-implant status over the years [1,12].

Since the available evidence remains fragmented, often limited to single product categories, precluding a comprehensive comparison across different inhaled tobacco and nicotine products, and also considering that the latest comprehensive studies date back to 2022 [12], an updated systematic synthesis of the evidence published after 2022 is warranted to clarify whether new evidence is available to bridge the aforementioned gaps in the literature.

Therefore, the aim of the present systematic review was to evaluate the effects of conventional cigarettes, heated tobacco products, electronic cigarettes, and waterpipe smoking on periodontal and peri-implant clinical and radiographic parameters, as well as on inflammatory biomarkers in gingival crevicular fluid and peri-implant sulcular fluid, in untreated adult users, in comparison with non-smokers.

## 2. Materials and Methods

### 2.1. Study Protocol

The study protocol was developed according to the principles of the Preferred Reporting Items for Systematic Reviews and Meta-Analyses (PRISMA) statement [14] (PRISMA checklist is available in Supplementary File S1) and is freely accessible on the International Prospective Register of Systematic Reviews (PROSPERO), where it was registered on 13 January 2026, before beginning the study selection process of records retrieved in the scientific literature (ID: CRD420261284851).

Based on the PECO model [15], the research question formulated for the present systematic review was as follows: “Are there differences in the clinical and radiographic periodontal and peri-implant status, as well as in the inflammatory biomarkers among untreated users of different inhaled tobacco and nicotine products compared with non-smokers?” In particular, the PECOs framework underlying this research question was as follows:

- P of Population: adults (≥18 years old) daily active users of inhaled tobacco and nicotine products;
- E of Exposure: use of inhaled tobacco and nicotine products, including traditional combustible cigarettes, heated tobacco products, electronic cigarettes, or waterpipe;
- C of Comparison: non-smokers of inhaled tobacco or nicotine products and comparisons among different smokers of inhaled tobacco and nicotine products;
- O of Outcomes: clinical and radiographical periodontal and peri-implant parameters (primary outcomes); inflammatory biomarkers in gingival crevicular fluid, or peri-implant sulcular fluid (secondary outcomes).

### 2.2. Search Strategy

The electronic databases of PubMed/MEDLINE, Scopus, and the Cochrane Library were searched by two independent reviewers (M.P.D.P. and F.D.S.) to retrieve English-language articles published between 25 April 2022 (date after the closing date of the search for the latest comprehensive systematic review [12]) and 23 December 2025.

In each database the refine to English language was the unique filter used and the following keywords relevant to the focus of the present systematic review were combined using Boolean operators: periodontal disease, periodontitis, peri-implant-disease, peri-implantitis, dental implant, implant loss, plaque index, gingival index, bleeding on probing, probing depth, tooth loss, missing teeth, marginal bone level, IL-1b, IL-8, IL-6, TNF-a, MMP-1, MMP-8, INF-y, IL-4, IL9, IL-10, IL-13, OPG, RANK-L, cigarette, e-cigarette, vaping cigarette, electronic cigarette, electronic nicotine delivery system, heat-not-burn tobacco, vape, vaping [12]. The full advanced search strategy for each database is provided in Appendix A.

The findings of the electronic search were implemented by conducting a manual search, screening the reference list of the articles included throughout the databases. The manual search was conducted by the aforementioned two independent reviewers (M.P.D.P. and F.D.S.).

### 2.3. Study Selection and Eligibility Criteria

Both for the records retrieved through the manual and electronic search, the study selection process was conducted by the two independent reviewers (M.P.D.P. and F.D.S.), who collected the retrieved records, removed duplicate records before screening the titles and abstracts, contacted the corresponding author of not available full-texts of the remaining records, and read the full-texts of potentially relevant records to exclude or include studies based on the eligibility criteria. A third reviewer (A.B.) was called at the end of each phase of the study selection process to resolve disagreements between the two independent reviewers (M.P.D.P. and F.D.S.) to proceed with the next phase.

An automation tool was used only to collect and manage the retrieved records: Mendeley Reference Manager, version 2.141.2.

Inclusion criteria were as follows: English clinical trials or observational studies published after 25 April 2022 and assessing clinical and/or radiographical periodontal and/or peri-implant status of different smokers of adults (≥18 years old), daily active smokers of inhaled tobacco or nicotine users, not undergoing any periodontal and/or peri-implant treatments.

Exclusion criteria were as follows: pre-clinical, in vitro, and review (any type) studies; conference papers, letters, books/chapters, and oral communication; studies published before 25 April 2022 or not in English; studies not reporting the in numerical quantitative value at least one (any) clinical or radiographic periodontal or peri-implant parameters; peri-implant parameters assessed during the biological healing period necessary to achieve osseointegration of 6 months after implant placement [16]; self-reported periodontal and/or peri-implant parameters; smokers with at least one of the following characteristics: <18 years old, former smokers, not daily smokers, smokeless tobacco, dual smokers (e.g., smokers both of traditional combustible cigarettes and electronic cigarettes), passive smokers, smokers who underwent any type of periodontal and/or peri-implant treatments.

### 2.4. Data Extraction and Collection

A standardized form for data extraction suggested for intervention reviews was compiled by two independent reviewers (M.P.D.P. and F.D.S.) [14], which extracted the following data:

- Studies: first author, year of publication, journal, reference, study design, quality, funding, aim/objective;
- Population: sample size, mean age, gender ratio;
- Periodontal/peri-implant status and outcomes: periodontal and peri-implant status, number of implants, time from implant placement/function, periodontal and peri-implant clinical, radiographic, and crevicular parameters, other parameters (e.g., missing teeth, salivary biomarkers);
- Main results (statistically significant);
- Authors’ conclusions.

A third reviewer (A.B.) was called after the data extraction for each study to resolve disagreements between the two independent reviewers (M.P.D.P. and F.D.S.) to proceed with the next included studies.

No corresponding authors of the included studies were contacted to request missing data or to validate reported data.

For eligible studies in which only a subset of the reported groups met the inclusion criteria, data were extracted exclusively for the relevant groups, while non-eligible groups were excluded from data extraction.

### 2.5. Data Synthesis

A qualitative synthesis of the data extracted and collected was performed. The synthesis focused on the characteristics of the study populations, different smokers of inhaled tobacco and nicotine products, and comparisons among groups of the primary and secondary outcomes. Descriptive statistical analyses were performed with the use of Microsoft Excel Software 2019 (Microsoft Corporation, Redmond, WA, USA) for the following purposes:

- Summarize the clinical and radiographic periodontal and peri-implant parameters reported among users of different inhaled tobacco and nicotine products and non-smokers;
- Compare periodontal and peri-implant clinical and radiographic outcomes across different inhaled tobacco and nicotine products and non-smokers;
- Summarize the inflammatory biomarkers assessed in gingival crevicular fluid or peri-implant sulcular fluid, and their reported associations with different inhaled tobacco and nicotine products;
- Compare the inflammatory biomarkers assessed in gingival crevicular fluid or peri-implant sulcular fluid across different inhaled tobacco and nicotine products and non-smokers;
- Identify patterns and trends in periodontal and peri-implant health parameters, as well as in inflammatory biomarker profiles according to the type of inhaled tobacco or nicotine products.

The results of the qualitative synthesis were structured and presented according to the type of inhaled tobacco and nicotine product.

For outcomes reported as continuous variables, pooled mean values were calculated as sample-size-weighted means of the study-level means, with each study mean weighted according to the corresponding number of participants contributing to that outcome. These pooled values were used for descriptive purposes and do not represent meta-analytic estimates.

To achieve the aim of assessing the peri-implant status in different smokers, a patient-level analysis of the outcomes related to dental implants was performed according to the 8th European Workshop on Periodontology, which encouraged patient-level analysis in cases in which disregarding data within patients may result in an underestimation of outcomes [16].

### 2.6. Risk of Bias Assessment

The Risk of Bias in Nonrandomized (ROBINS-I) tool [17] was used to assess the risk of bias and the overall study’s quality by two independent reviewers (M.P.D.P. and F.D.S.). A third reviewer (A.B.) was called after the risk assessment for each study to resolve disagreements between the two independent reviewers (M.P.D.P. and F.D.S.) before proceeding with the next included study.

ROBINS-I was used to frame the research question with reference to a hypothetical target trial comparing different inhaled tobacco and nicotine products versus non-use on periodontal and peri-implant outcomes.

Domains were operationalized as follows: confounding was judged according to whether key periodontal and peri-implant confounders (age, gender, comorbidities, implant-related factors, etc.) were adequately controlled or balanced; exposure classification considered the clarity and consistency of definitions of daily active use of conventional cigarettes, heated tobacco products, electronic cigarettes, or waterpipe, and the exclusion of dual/former/passive users as specified in the eligibility criteria; participant selection focused on consecutive or clearly defined sampling and the risk of selection bias related to periodontal/peri-implant status; outcome measurement assessed the validity and consistency of clinical (e.g., probing depth, clinical attachment level, bleeding on probing) and radiographic parameters and of biomarker assays; missing data and selective reporting were evaluated based on completeness of reported outcomes and any evidence of selective non-reporting.

## 3. Results

### 3.1. Study Selection

The titles of the records retrieved through the electronic search (*n* = 944, of which 667 were from PubMed/MEDLINE, 190 from Scopus, and 87 from Cochrane Library) were screened, and the duplicates were removed (*n* = 116). Reading the titles and abstracts of the remaining records (*n* = 828) led to the elimination of ineligible records (*n* = 756).

Three full-text records were unavailable for assessment, and the corresponding authors who were contacted did not respond to requests for access. Consequently, these records (*n* = 3) were excluded due to the unavailability of full texts. Reading the full texts of the remaining records (*n* = 69) led to the elimination of studies that were not compliant with at least one inclusion/exclusion criterion (*n* = 40), while the remaining records were included studies from the electronic search (*n* = 28) [18,19,20,21,22,23,24,25,26,27,28,29,30,31,32,33,34,35,36,37,38,39,40,41,42,43,44,45].

The same procedure for study selection was conducted for the manual search in the bibliography of the previously included studies [18,19,20,21,22,23,24,25,26,27,28,29,30,31,32,33,34,35,36,37,38,39,40,41,42,43,44,45].

The titles of the references retrieved through the manual search (*n* = 1086) were screened, and the duplicates were removed (*n* = 190). Reading the titles and abstracts of the remaining records (*n* = 896) led to the elimination of ineligible records (*n* = 876).

One full-text record was unavailable for assessment, and the corresponding author who was contacted did not respond to requests for access. Consequently, this record (*n* = 1) was excluded due to the unavailability of the full text. Reading the full texts of the remaining records (*n* = 19) led to the elimination of studies that were not compliant with at least one inclusion/exclusion criterion (*n* = 16), while the remaining records were included studies from the manual search (*n* = 3) [46,47,48].

Combining the records included from the electronic search and the manual search, 31 records were included in this systematic review [18,19,20,21,22,23,24,25,26,27,28,29,30,31,32,33,34,35,36,37,38,39,40,41,42,43,44,45,46,47,48].

Table 1 provides the list of excluded studies, both from the electronic and manual search, and the related justification for the exclusion.

**Figure 1:**
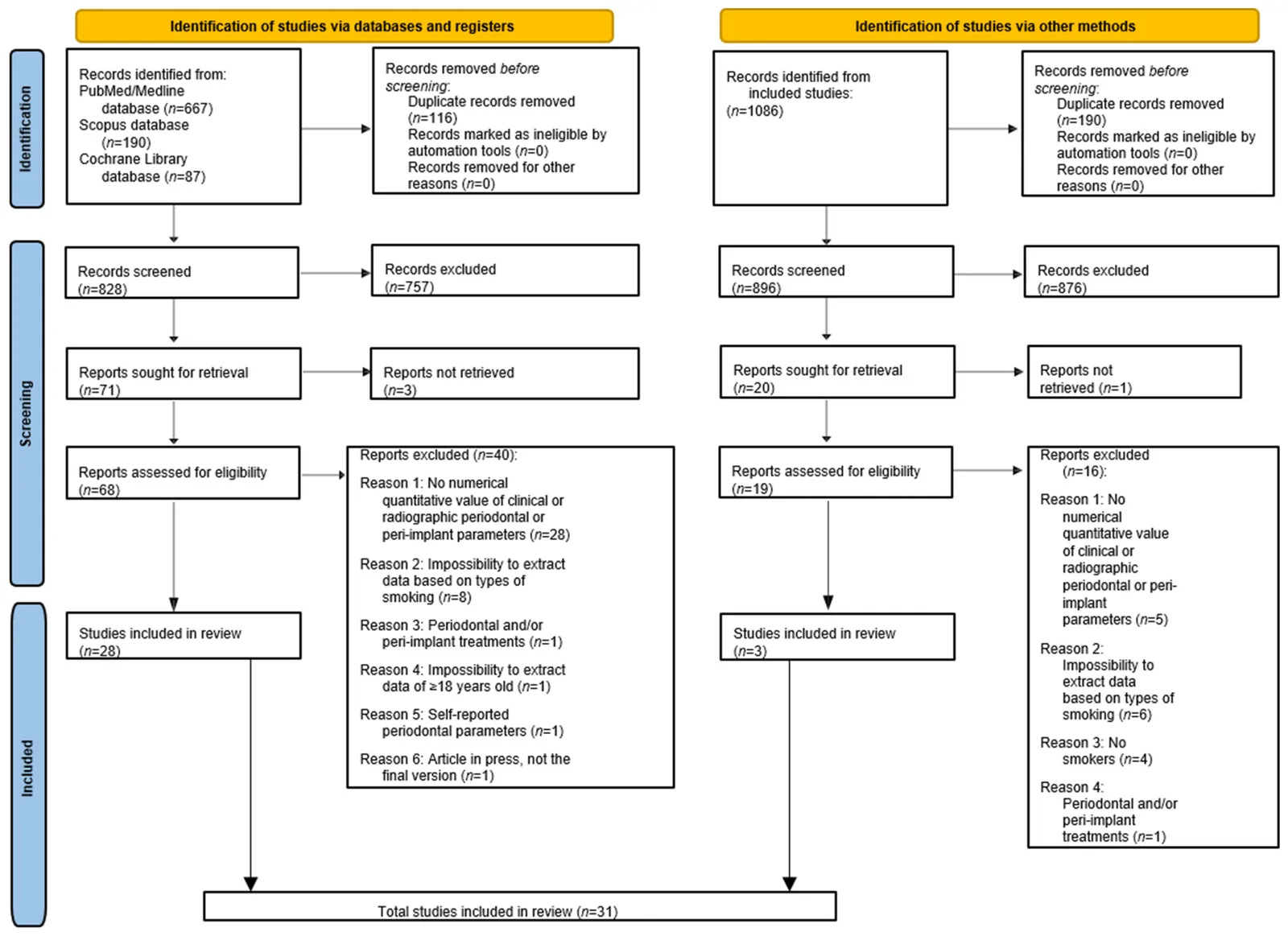
PRISMA 2020 flowchart illustrating the selection pathway of studies from initial identification to final inclusion, conducted in accordance with the PRISMA 2020 statement.

Figure 1 summarizes the selection pathway of studies from initial identification to final inclusion, conducted in accordance with the PRISMA 2020 statement.

### 3.2. Study Characteristics

A total of 31 studies [18,19,20,21,22,23,24,25,26,27,28,29,30,31,32,33,34,35,36,37,38,39,40,41,42,43,44,45,46,47,48] (26 cross-sectional studies [18,19,21,22,23,24,25,26,27,28,29,30,31,32,33,35,37,39,40,41,42,43,44,46,47,48], two case-control studies [20,36], one retrospective study [45], one prospective cohort study [38], one case-control cross-sectional study [34]) including 3106 subjects were extracted. Table 2 shows the characteristics of the 31 included studies [18,19,20,21,22,23,24,25,26,27,28,29,30,31,32,33,34,35,36,37,38,39,40,41,42,43,44,45,46,47,48], the related main results and conclusions.

### 3.3. Data Extraction and Synthesis

#### 3.3.1. Periodontal Status Among Different Users of Inhaled Tobacco and Nicotine Products

A total of 23 studies [19,20,23,24,26,27,28,29,31,32,34,35,36,37,39,40,41,42,43,44,46,47,48] including 2447 subjects investigated the periodontal status in different users of Inhaled Tobacco and nicotine products. In particular, 23 studies [19,20,23,24,26,27,28,29,31,32,34,35,36,37,39,40,41,42,43,44,46,47,48] of non-smokers, 21 studies [19,20,23,24,26,27,29,31,32,34,35,36,37,39,40,41,42,43,44,47,48] of traditional combustible cigarettes, seven studies [20,24,28,29,35,37,46] of electronic cigarettes, two studies [32,39] of heated tobacco products, and two studies [19,27] of waterpipe.

Periodontal clinical, radiographical, and inflammatory parameters among different users of inhaled tobacco and nicotine products are clustered in Table 3.

#### 3.3.2. Peri-Implant Status Among Different Users of Inhaled Tobacco and Nicotine Products

A total of eight studies [18,21,22,25,30,33,38,45] including 659 subjects investigated the peri-implant status in different users of inhaled tobacco and nicotine products. In particular, eight studies [18,21,22,25,30,33,38,45] of non-smokers, eight studies [18,21,22,25,30,33,38,45] of smokers of traditional combustible cigarettes, one study [45] of unfiltered cigarette smokers, and one study of waterpipe [21].

Peri-implant clinical, radiographical, and inflammatory parameters among different users of inhaled tobacco and nicotine products are clustered in Table 4 following a patient-level analysis.

### 3.4. Risk of Bias and Quality Assessment

Ten studies [19,20,25,28,30,34,35,36,38,39] were found to have an overall low risk of bias, 10 studies [18,21,22,26,29,33,40,44,45,48] a moderate risk of bias, seven studies [23,24,31,32,43,46,47] a serious risk of bias, and four studies [27,37,41,42] a critical risk of bias. Table S1 in Supplementary File S2 shows the risk of bias assessment item-by-item and the related overall quality.

## 4. Discussion

The present systematic review aims to evaluate and compare the effects of conventional cigarettes, heated tobacco products, electronic cigarettes, and waterpipe smoking on periodontal and peri-implant clinical and radiographic parameters, as well as on inflammatory biomarkers in gingival crevicular fluid.

A total of 31 studies, comprising 3106 subjects, were included.

### 4.1. Study Population

#### 4.1.1. Traditional Combustible Cigarette Smokers

The World Health Organization (WHO) showed in the latest “WHO global report on trends in prevalence of tobacco use 2000–2024 and projections 2025–2020” that, despite the decline in smoking rates, one in every five adults continues to use tobacco [105]. The total number of tobacco smokers between 2000 and 2024 fell to 1.2 billion from 1.38 billion in 2020 [106]. However, in its report, the WHO combines data on smokers of traditional cigarettes with combustible cigarettes with alternatives based on tobacco without combustion [105], thus possibly underestimating the progress made in smoking cessation among traditional adult smokers who are trying to quit.

As expected, the group of traditional combustible cigarette smokers (CS) was the most extensively represented in the present systematic review compared to other categories of smokers, both in analyses addressing periodontal conditions and those focusing on peri-implant status.

Among CS evaluated for periodontal outcomes, the mean age showed a noticeable variability across subgroups. While the overall periodontal population consisted predominantly of middle-aged adults (overall mean age: 39.97 ± 11.61 years), the subgroup characterized by periodontal health presented a substantially younger mean age (29.12 ± 9.32 years). This age difference likely reflects the natural history of periodontal disease, which, without interference, progresses at relatively continuous rates [107], as well as may reflect variations in smoking exposure and cumulative risk over the years [108].

In contrast, such age-related imbalances were not observed among participants assessed for peri-implant outcomes. In this group, CS exhibited a consistently higher mean age (overall mean: 52.55 ± 5.82 years), with no relevant differences between peri-implant status subgroups.

#### 4.1.2. Waterpipe Smokers

In the present systematic review, waterpipe smokers (WS) were represented by a limited number of subjects (*n* = 62 for periodontal outcomes; *n* = 24 for peri-implant outcomes) and investigated in a limited number of studies (two [19,26] and one [21], respectively), all recruited in Kuwait and Saudi Arabia. The mean age was 37.27 ± 9.01 years for periodontal and 53.8 ± 2.4 years for peri-implant evaluation. The geographical distribution and the mean age are in line with the history of waterpipe, which was introduced in Asia in the 16th century for adult use [109]. The recent trend shows waterpipe usage spreading among younger adults and in Western regions [109]. However, this trend has not yet been followed by scientific periodontal research.

#### 4.1.3. Heated Tobacco Products Smokers

Heated tobacco products (HTP) were marked as less harmful compared with CS due to the lower emissions of chemical products (up to 95% lower than CS) [110]. Sun et al. (2023) [110] estimated for the first time the prevalence of HTP users worldwide, reporting a pooled global prevalence of HTP users at 4.87% for lifetime use [110]. In most countries analyzed, daily HTP users remained below 2%, except in the Czech Republic, Cyprus, Italy, Poland, and Slovakia [110]. The highest pooled prevalence of lifetime HTP use was identified in the Western Pacific and European regions, where an increasing trend was documented between 2015 and 2020 [110].

Despite the growing diffusion of HTPs, evidence regarding their effects on periodontal and peri-implant tissues remains scarce. Only two studies [32,39] included in the present systematic review investigated the periodontal status of HTP users, and none focused on peri-implant status. Both of these studies [32,39] were conducted in a European setting, specifically at the Clinical Hospital Center Rijeka in Croatia, and were based on a limited overall sample size comprising only 45 HTP users.

When interpreting data relating to HTP users, particular attention must be paid not only to the small sample size but also to the gender distribution of the study population. In fact, in the present systematic review, HTP users were the only group showing a higher prevalence of females compared with males, with a male-to-female ratio of 1:3.09. This imbalance was recognized as a potential bias in the original study by Božac et al. [32], as it is unlikely to reflect the gender distribution of HTP users in the general population. Indeed, these findings contrast with the results of the meta-regression analysis by Sun et al. [110], which reported a higher prevalence of current HTP users among males than in females.

#### 4.1.4. Electronic Cigarette Smokers

Electronic cigarettes (E-Cigs) are an effective tool to quit smoking and are reported as less harmful compared with CS, even if not risk-free [111]. The WHO global report estimated the use of E-Cigs worldwide for the first time, registering that there are more than 100 million E-Cig users [106]. At least 86 million are adults, especially from high-income countries, and 15 million are children between 13 and 15 years of age [106]. There is a constant increase in E-Cig users, in particular among children and young adults, probably due to the at least 16,000 attractive flavors available and the cartoon designs of the product boxes [112]. Furthermore, electronic cigarette content on social media is associated with more positive attitudes compared with other smoking products [112]. Even in the present systematic review, despite the exclusion of subjects younger than 18 years, E-Cigs exhibited a lower mean age (32.49 ± 5.65 years) compared with all other smoking categories. In addition, the E-Cig group showed the highest male-to-female ratio of 11.73:1.

An interesting consideration is the geographic distribution of the included studies evaluating periodontal status in E-Cigs. Of the seven studies included, four were conducted in the Middle Eastern (Saudi Arabia) and Asian countries (Kuwait, Malaysia, and China). This geographic distribution does not reflect the highest prevalence of E-Cigs users reported in the WHO data, which is observed in the WHO European Region, where adult E-Cigs users reach 4.6%, more than double the global average [106]. This discrepancy highlights the need to extend periodontal research on E-Cigs to countries with higher prevalence.

Unfortunately, no study investigating peri-implant status in E-Cig users was included in the present systematic review.

### 4.2. Clinical Periodontal and Peri-Implant Status Among Different Users of Inhaled Tobacco and Nicotine Products

Overall, the clinical periodontal findings of the present systematic review indicate a worse periodontal status among smokers compared with NS, with CS exhibiting the most severe clinical periodontal status.

Across the majority of included studies, CS showed significantly higher plaque-related indices, probing pocket depth (PPD), clinical attachment loss (CAL), and tooth loss compared with NS, confirming smoking as a major modifier of periodontal disease severity [4]. These findings were consistently reported regardless of periodontal status and were further reinforced in studies specifically addressing generalized periodontitis, where CS exhibited statistically significantly deeper PPD and greater CAL than NS [34,35,44].

Interestingly, plaque accumulation was higher in CS than in NS, regardless of the indices used for assessment (plaque index, modified plaque index (mPI), full mouth plaque score), suggesting that smoking may impair plaque control or alter plaque composition, inducing oral microbiome dysbiosis and probably making smokers more prone to severe periodontal clinical status [113].

As expected, despite higher plaque accumulation and greater tissue destruction, inflammatory clinical signs such as the gingival index (GI) and bleeding on probing (BoP) were frequently statistically lower in CS compared with NS [31,48]. These findings are in line with the well-documented vasoconstrictive quantitative redistribution effects of nicotine on small and large vessels of the superficial and deeper gingival papilla connective tissue, which attenuate clinical signs of inflammation while periodontal disease progression continues subclinically [114].

E-Cigs generally exhibited an intermediate periodontal clinical status, with periodontal parameters often worse than NS but less severe than CS. Several studies reported significantly higher PPD and CAL in E-Cigs users compared with NS [20,28,37], indicating that vaping is not clinically safe for periodontal tissues. As for CS, the plaque-related parameters in E-Cigs were generally higher compared with NS, but comparable to CS. Beyond these quantitative findings on plaque accumulation, it is also interesting to consider qualitative knowledge when interpreting other periodontal data on E-Cigs. In particular, Thomas et al. [115] identified a unique microbiome in E-Cigs, with similarities to the microbiomes of CS and NS, and in particular with a significantly greater affinity to that of CS. Thomas et al. [115] therefore hypothesized that there is a unique periodontal risk associated with the use of e-cigarettes.

In association with that, notably, community periodontal index-based assessments and treatment needs were significantly worse in E-Cigs compared with NS [29], supporting that E-Cigs may contribute to periodontal disease, although CS reported more complicated periodontal treatment needs.

Contrary to expectations and the results of the previous systematic review [12], the BoP (%) was higher among E-Cigs (53.0 ± 18.5%) compared with NS (37.8 ± 17.5%), and was similar to HTP users (50.78%), despite the potential effect of vasoconstriction by nicotine. However, the GI values were lower among NS (0.414 ± 0.27) and E-Cigs (1.15 ± 0.03).

Data on HTP users were limited but suggested a more favorable periodontal profile compared with CS. Mišković et al. [39] reported significantly lower PPD in HTP users compared with CS, while Božac et al. [32] showed plaque levels in HTP users comparable to CS but not significantly different from NS. These findings, although preliminary, reinforce the previous hypotheses that HTPs may be associated with a less harmful clinical impact on the periodontium compared with CS [39]. However, the small sample size and limited number of studies prevent definitive conclusions. Further studies on a larger sample size should confirm the potentially positive effect of HTP compared with CS, which, despite having a negative effect, could be an alternative in smoking cessation programs, considering also that a previous study on CS switching to IQOS^®^ demonstrated improvement in clinical periodontal disease parameters after 6 months [116].

WS demonstrated clinical periodontal parameters comparable to CS, particularly with respect to gingival inflammation, probing depth, and tooth loss. Almeslet et al. [26] reported significantly worse PI, GI, PPD, and the number of missing teeth in WS compared with NS.

Concerning the peri-implant clinical outcomes, findings indicate a negative effect of smoking on peri-implant tissues, with CS and WS showing the worst peri-implant inflammatory and plaque-related indices compared with NS.

Plaque-related indices (mPI, full mouth plaque index) were markedly higher in CS compared with NS across all peri-implant conditions, including peri-implant health, mucositis, and peri-implantitis. In the pooled analysis, mPI values were more than doubled in CS (1.53 ± 1.06) compared with NS (0.645 ± 0.60), with WS showing the highest plaque accumulation (2.4 ± 0.2). These findings were confirmed by individual studies, which reported significantly higher mPI in CS and WS compared with NS, regardless of peri-implant status [21,30,33]. Despite higher plaque accumulation and deeper peri-implant probing depth (PD), inflammatory clinical signs (mGI and BoP) were lower in CS than in NS, especially in peri-implantitis subgroups (0.67 ± 0.43 vs. 1.69 ± 1.03 for mGI, and 35.7 ± 14.6% vs. 28.5 ± 7.8%, respectively). This pattern is similar to that in periodontal tissues and is explained by the smoking-induced reduction in the inflamed dental implant in bleeding after gentle probing [117].

PD emerged as one of the most consistently affected peri-implant parameters. Mean PD values were substantially higher in CS (4.30 ± 0.77 mm) and WS (4.5 ± 0.4 mm) compared with NS (2.68 ± 0.41 mm). Several studies confirmed significantly higher PD in CS and WS compared with NS [21,25,33], and PD was also positively correlated with microbial dysbiosis, including oral yeast levels, in CS with peri-implantitis [22].

### 4.3. Radiographic Periodontal and Peri-Implant Status Among Different Users of Inhaled Tobacco and Nicotine Products

With respect to the radiographic periodontal status, the findings of the present systematic review indicate that CS is consistently associated with increased marginal bone loss (MBL) around natural teeth when compared with NS. In particular, studies by Ali et al. [19] and Almeslet et al. [26] demonstrated significantly higher MBL, along with worse clinical periodontal parameters, among CS compared with periodontally healthy non-smokers (*p* < 0.001 and *p* < 0.05, respectively). Similarly, Almeslet et al. [26] exhibited significantly greater MBL among WS compared with NS.

Notably, E-Cigs also showed markedly increased MBL compared with NS, and to a smaller extent with CS. Both Ali et al. [19] and Alnufaiy et al. [28] reported significantly higher MBL in E-cigarette users (*p* < 0.05), a finding that is corroborated by the extracted overall MBL mean values, which revealed the highest MBL among E-Cigs compared with all smoking categories. These results are in contrast with the previous meta-analysis of Shabil et al. [118], which reported no significant difference in MBL between NS and E-Cigs. Notably, the most recent studies included in the meta-analysis by Shabil et al. [118] reported effect estimates that were higher than the overall mean, while the majority of studies published before 2020 reported lower values compared with the mean. This temporal pattern may partially account for the discrepancies observed between the findings of the present systematic review and those reported by Shabil et al. [118], suggesting a potential dose-dependent effect of E-Cig exposure. Further analyses stratified by E-Cig smoking intensity and duration may explain these differences.

Furthermore, the presence of systemic modifiers such as prediabetes appeared to exacerbate the MBL in CS. Almeslet et al. [27] reported significantly higher mesial and distal MBL in CS with prediabetes compared with NS, both with and without prediabetes. These findings are in line with the recent results of Hua et al. (2025) [119], which found that CS and periodontitis synergistically augmented the risk of prediabetes, and the negative effect of periodontitis on glycemic status could be reduced by smoking cessation.

With respect to the radiographic peri-implant status, the findings of the present systematic review indicate significantly greater MBL in CS and WS compared with NS, both in peri-implant health and disease. Smoking-related differences in MBL were most evident in the peri-implantitis subgroup. This finding is not unexpected, as peri-implant health is by definition characterized by limited bone loss, not exceeding 2 mm [3]. Nevertheless, interestingly, even within the peri-implant health subgroup, CS exhibited MBL values approaching the diagnostic threshold, whereas NS consistently showed substantially lower values. This pattern suggests that CS may predispose peri-implant bone remodeling [120]. Studies by Ahmed et al. [18], Dewan et al. [33], and Aljudaibi et al. [22] showed significantly higher MBL in CS with peri-implantitis compared with NS with peri-implantitis or peri-implant health (*p* < 0.05–0.01). Obviously, peri-implantitis status consistently exhibited the greatest MBL regardless of smoking status, with CS showing the most severe values.

Longitudinal studies further support the negative effect of CS on peri-implant MBL. Tanik et al. [45] demonstrated a significant increase in MBL over time in CS and unfiltered cigarette smokers. At four years post-implant placement, both CS and unfiltered cigarette smokers exhibited significantly higher MBL compared with NS (*p* < 0.001) [45]. These findings indicate that while marginal bone remodeling occurs physiologically after implant placement, smoking appears to accelerate and exacerbate this process [120].

Unfortunately, no evidence regarding the radiographical status in HTPs was reported in the included studies, while the peri-implant radiographical status was even more limited to the comparison between NS and CS.

### 4.4. Inflammatory Biomarker Among Different Users of Inhaled Tobacco and Nicotine Products

The included studies consistently demonstrated a smoking-related modulation of inflammatory biomarkers in both saliva and gingival crevicular fluid.

CS consistently exhibited the most pronounced pro-inflammatory and tissue-destructive biomarker profile across both periodontal and peri-implant studies. Several Authors reported significantly increased levels of pro-inflammatory cytokines, including interleukin (IL)-1β, IL-8, IL-15, IL-18, tumor necrosis factor (TNF)-α, and prostaglandin E2 in CS compared with NS [19,20,21,26,30,37]. These cytokines are known to promote leukocyte recruitment, osteoclast activation, and connective tissue breakdown, and their elevation is considered detrimental to periodontal and peri-implant tissue [12,121,122,123,124].

Neutrophil-related biomarkers, such as citrullinated histone H3, neutrophil elastase, calprotectin, and myeloperoxidase, were also significantly higher in CS with periodontitis compared with NS with periodontitis [36]. These markers reflect neutrophil hyperactivation, key cells against foreign pathogens, and the formation of neutrophil extracellular traps, processes associated with tissue damage [125,126]. The pathogenesis of periodontitis involves an immune-inflammatory reaction in which impaired neutrophil extracellular trap formation and/or elimination exacerbates the inflammatory reaction and the related periodontal tissue destruction [125]. The positive correlations observed between these biomarkers and BoP or CAL further support their pathological and inflammatory role [36].

Smoking-related alterations in antimicrobial peptides were reported by Soldati et al. [43], who showed significantly lower β-defensin 1 and higher β-defensin 2 levels in periodontal sites of CS with periodontitis compared with NS. While β-defensins contribute to microbial control, their dysregulated expression in CS may reflect the negative effect of smoking on the neutrophil defense mechanism [127].

Bone metabolism markers were also affected by CS. Gajendran et al. [34] reported significantly higher cathepsin K levels in CS, a protease involved in bone resorption, with a positive correlation with RANKL observed only in CS. This finding indicates enhanced osteoclast activity and supports the detrimental effect of CS on marginal bone [128,129].

E-Cigs showed a mixed inflammatory profile, generally intermediate between NS and CS. Li et al. [37] reported significantly lower salivary IL-8 and IL-6 levels in E-Cigs compared with CS and NS, which may suggest a reduced chemotactic and acute inflammatory response. However, Ali et al. [20] observed significantly higher salivary IL-15 and IL-18 levels in E-Cigs compared with non-smokers. These cytokines are involved in T-cell and natural killer cell activation, and their increase may indicate persistent immune stimulation [130].

Alnufaiy et al. [28] reported higher salivary IL-1β levels in E-Cigs compared with NS in bivariate analysis, although this difference was not confirmed after multivariate adjustment, suggesting potential confounding factors. Overall, while some inflammatory markers appear reduced in E-Cigs users compared with CS, the elevation of key cytokines involved in chronic inflammation indicates that E-Cigs are not biologically inert for periodontal tissues.

WS demonstrated a pro-inflammatory profile comparable to CS for specific biomarkers. Ali et al. [19,21] reported significantly higher concentrations of advanced glycation end products (AGEs) in WS compared with NS in both periodontal and peri-implant studies. AGEs promote oxidative stress, impair collagen turnover, and enhance inflammatory signaling through activation of their receptors (RAGE) [131,132].

Similarly, Almeslet et al. [26] reported significantly higher salivary prostaglandin E2 levels in WS compared with NS. Prostaglandin E2 plays a key role in vasodilation and pain perception, and its increased levels are associated with periodontal inflammation [26,133]. Despite the limited number of WS included in the studies, these findings suggest that waterpipe smoking may induce inflammatory responses comparable to those observed in CS.

NS generally exhibited a more balanced inflammatory profile, characterized by lower levels of pro-inflammatory cytokines and tissue-destructive enzymes. Soldati et al. [43] showed that periodontally healthy non-smokers had significantly higher β-defensin 1 levels, indicating effective innate antimicrobial defense. In addition, site-specific increases in β-defensin 1 and 2 were observed in NS with periodontitis but not in smokers, suggesting a preserved local immune responsiveness.

Taskaldiran et al. [48] reported lower IL-17 levels in NS with periodontitis compared with smokers, while IL-35 levels were lower in periodontally healthy NS than in smokers and diseased NS. IL-17 is a pro-inflammatory cytokine involved in neutrophil recruitment and bone/connective tissue destruction [134], while IL-35 is an anti-inflammatory cytokine associated with Treg immune suppression, which plays a protective role in periodontal disease [135,136]. These results support the hypothesis that IL-17 and IL-35 have a significant role in periodontitis pathogenesis, and that this role may be altered by smoking habits.

In peri-implant tissues, CS was consistently associated with increased levels of HMGB-1, IL-1β, TNF-α, soluble urokinase plasminogen activator receptor, and MMP-9, particularly in peri-implantitis [18,30,33]. These biomarkers are involved in inflammatory amplification, extracellular matrix degradation, and osteoclastic activation [137,138]. Their elevation was correlated with clinical parameters such as BoP and PD, supporting their role as markers of disease severity [18,33].

WS also exhibited significantly higher peri-implant AGE levels compared with NS [21], suggesting increased oxidative and inflammatory stress around implants [139,140,141]. Collectively, these findings indicate that smoking-related exposure, regardless of the delivery system, negatively affects peri-implant tissue homeostasis, with conventional cigarettes exerting the strongest and most consistent effect.

Unfortunately, no evidence regarding the inflammatory biomarker status in HTPs was reported in the included studies, while the peri-implant inflammatory status was limited to the comparison between NS and CS, and limited to the level of AGEs for WS only.

### 4.5. Strengths, Limitations, and Future Prospects

The main strength of the present systematic review is its comprehensive and comparative approach, as it simultaneously evaluated the effects of different types of inhaled tobacco and nicotine delivery systems, including CS, E-Cigs, HTP users, and WS, both on periodontal and peri-implant clinical, radiographic, and inflammatory outcomes. In particular, compared to the previous systematic review [12], the present study includes data on HTP users for the first time.

However, most included studies were cross-sectional, precluding the study of disease progression over time in the different types of inhaled tobacco and nicotine delivery systems. Additionally, a key limitation across the primary literature pertains to potential confounding. Critical covariates, such as participant age, gender, socioeconomic status, systemic health conditions (e.g., diabetes mellitus), duration and daily intensity of product use, exert a major influence on periodontal and peri-implant health. While some studies performed multivariate adjustments, many provided unadjusted univariate analyses or lacked robust control for these variables; consequently, residual confounding cannot be ruled out and may substantially account for or modulate the observed clinical and biomarker differences. Moreover, some smoking categories, particularly HTP users and WS, were investigated in a limited number of studies with small sample sizes and restricted geographic representation. Furthermore, within the different categories of smokers, the periodontal/peri-implant parameters evaluated were different, creating greater heterogeneity, which precluded the opportunity to quantitatively analyze data. A complete lack of data was unfortunately found for peri-implant outcomes of HTP and E-Cigs users, representing a significant knowledge gap in the literature.

Furthermore, in accordance with our eligibility criteria, dual users were excluded. Although this methodological choice was essential to investigate the specific profile of smokers attributable to individual delivery systems without overlapping exposure categories, it presents a key limitation regarding external validity. Dual use represents a consumption pattern among real-world nicotine users, which requires further independent evaluation. Consequently, while the present findings clarify product-specific periodontal and peri-implant status, they cannot be directly generalized to real-world populations of dual or poly-nicotine users.

Finally, it should be emphasized that the pooled values reported in Table 3 and Table 4 represent sample-size-weighted descriptive aggregates of study-level means rather than meta-analytic estimates. Although weighting by sample size reduces the influence of smaller studies, these summary values remain subject to the substantial clinical and methodological heterogeneity across the included studies. Therefore, apparently higher or lower pooled values for specific products should be interpreted cautiously, in particular for the HTP and WS groups, for which periodontal outcomes were reported in only two studies, resulting in limited evidence and reduced robustness of the corresponding descriptive estimates. Accordingly, findings such as the higher pooled MBL observed among E-Cig users or the higher pooled BoP% observed among E-Cig users compared with conventional smokers with periodontitis should be regarded as descriptive patterns that require confirmation in larger, well-designed comparative studies rather than as definitive evidence of differences between products.

The presence of serious-to-critical risk of bias in a substantial proportion of studies (11 of the 31 included) further limits the strength of the conclusions. These studies were retained in the qualitative synthesis but were interpreted with greater caution. Given the predominantly qualitative nature of the synthesis and the heterogeneity of the included studies, a formal sensitivity analysis excluding these higher-risk studies was not performed. Nevertheless, the overall conclusions of the review are based on the full body of evidence and take into account the limitations related to risk of bias.

Based on the aforementioned limitations, future research should focus on longitudinal and prospective designs to clarify temporal and dose–response relationships between different smokers of inhaled tobacco and nicotine delivery systems and periodontal and peri-implant disease progression. Particular focus should be placed on emerging nicotine delivery systems, such as E-Cigs and HTP, especially in relation to peri-implant tissues.

## 5. Conclusions

The present systematic review provides an updated overview of the effects of different inhaled tobacco and nicotine products on periodontal and peri-implant status.

CS remains the type of smoking associated with the most consistently worsened periodontal and peri-implant outcomes, such as higher plaque accumulation, deeper PPD, greater CAL, increased MBL, and more missing teeth. CS also induces a pro-inflammatory biomarker profile in both periodontal and peri-implant tissues, confirming its role as a major modifiable risk factor.

E-Cigs and HTPs appear to have less harmful effects; however, this pattern was not consistent across all outcomes. E-Cigs users often show worse periodontal parameters than NS, particularly in PPD and CAL, while inflammatory parameters such as BoP are not reduced, suggesting a persistent inflammatory environment despite lower GI compared with NS. Importantly, the radiographic findings represent a notable exception to the generally intermediate profile of E-Cigs: E-Cig users showed the highest overall MBL values among all smoking categories, exceeding those observed in CS. Although the available radiographic evidence is limited, this finding suggests that E-Cig use may have a relevant adverse effect on alveolar bone levels that is not fully reflected by the overall clinical periodontal profile. HTPs showed slightly more favorable periodontal status compared with CS, but the data are preliminary due to restricted sample size.

WS demonstrates clinical and radiographical parameters generally comparable to CS, underscoring that it is not a safe alternative.

Overall, while alternative nicotine products may be associated with less harmful effects than CS, none are risk-free for periodontal and peri-implant status. In fact, the available evidence does not support considering them consistently less harmful across all outcomes. In particular, the elevated MBL observed among E-Cig users warrants caution and further investigation. Moreover, the available evidence suggests that some periodontal parameters may be less adversely affected among E-Cig and HTP users than among conventional cigarette smokers. However, these findings should be interpreted cautiously given the limited number of studies, small sample sizes, and substantial heterogeneity across populations, exposure characteristics, and periodontal outcomes. Importantly, the available evidence remains insufficient to establish the long-term periodontal or peri-implant safety of E-Cigs or HTPs.

The heterogeneity of current evidence, small sample sizes, and lack of longitudinal data should be taken into account in the interpretation of conclusions, especially for E-Cigs and HTPs.

Future prospective studies are needed to clarify the long-term effects of emerging nicotine delivery systems.

## Figures and Tables

**Table 1:** List of excluded studies, both from the electronic and manual search (indicated with the symbol “^‡^”), and the related justification for the exclusion.

Justification for the Exclusion	Studies Excluded (*n* = 56)	Number
No numerical quantitative value of clinical or radiographic periodontal or peri-implant parameters	Alamer et al., 2024 [49]; Alharethi et al., 2024 [50]; ^‡^ Al-Kubaisi et al., 2023 [51]; ^‡^ AlMubarak et al., 2022 [52]; AlQobaly et al., 2022 [53]; Alrashdan et al., 2025 [54]; Antonello et al., 2023 [55]; Chido-Amajuoyi et al., 2022 [56]; ^‡^ Bhargave et al., 2022 [57]; Cirano et al., 2024 [58]; Conte et al., 2023 [59]; da Silva et al., 2023 [60]; Guarnieri et al., 2024 [61]; Han et al., 2022 [62]; Harrandah et al., 2024 [63]; Hashimoto et al., 2025 [64]; He et al., 2025 [65]; Herrera-Serna et al., 2025 [66]; Huang et al., 2024 [67]; Kowalski et al., 2025 [68]; Kumar et al., 2024 [69]; Miluna et al., 2022 [70]; Miluna-Meldere et al., 2024 [71]; Park et al., 2023 [72]; Prince et al., 2024 [73]; Rahman et al., 2023 [74]; Rashid et al., 2024 [75]; ^‡^ Revas et al., 2024 [76]; Sánchez et al., 2024 [77]; ^‡^ Sever et al., 2023 [78]; Soares et al., 2023 [79]; Vu et al., 2023 [80]; Yang et al., 2025 [81]	Total: *n* = 33 (28 from electronic and 5 from manual search)
Impossibility of extracting data based on types of smoking	^‡^ Adam et al., 2024 [82]; Aghasizadeh et al., 2024 [83]; Al Naasan et al., 2022 [84]; ^‡^ Ayele et al., 2023 [85]; Costa et al., 2025 [86]; Eger et al., 2025 [87]; Farina et al., 2025 [88]; ^‡^ Halstenbach et al., 2023 [89]; ^‡^ Isola et al., 2022 [90]; Laniado et al., 2022 [91]; ^‡^ Martinez-Amargant et al., 2023 [92]; ^‡^ Mauland et al., 2025 [93]; Testori et al., 2022 [94]; Trullenque-Eriksson et al., 2023 [95]	Total: *n* = 14 (8 from electronic and 6 from manual search)
No smokers	^‡^ Abood et al., 2023 [96]; ^‡^ Al-Daragi et al., 2024 [97]; ^‡^ Aldosari et al., 2023 [98]; ^‡^ Mohammed et al., 2022 [99]	Total: *n* = 4 (4 from manual search)
Periodontal and/or peri-implant treatments	Amerio et al., 2025 [100]; ^‡^ Tamashiro et al., 2023 [101]	Total: *n* = 2 (1 from electronic and 1 from manual search)
Impossibility of extracting data for those ≥18 years old	Khattak et al., 2024 [102]	Total: *n* = 1 (1 from electronic search)
Self-reported periodontal parameters	Mohajeri et al., 2023 [103]	Total: *n* = 1 (1 from electronic search)
Article in press, not the final version	David et al., 2025 [104]	Total: *n* = 1 (1 from electronic search)

**Table 2:** Studies characteristics of the included records in the present systematic review: first author, year of publication, journal, reference, study design, quality, funding, aim/objective; population sample size, mean age, gender ratio, number of implants; periodontal and peri-implant status, time from implant placement/function, periodontal and peri-implant clinical, radiographic, and crevicular parameters, other parameters (e.g., missing teeth, salivary biomarkers), main results (statistically significant); conclusions.

First Authors, Year Journal, Reference Study Design Quality Funding Aim/Objective	Population Sample Size Smoking Behavior Mean Age Gender Ratio (M:F) Number of Implants	Periodontal/Peri-Implant Status at Baseline Time Points Clinical, Radiographical, and Crevicular Parameters Other Parameters	Main Results (Statistically Significant)	Conclusions
Ahmed A.R., 2022 Eur Rev Med Pharmacol Sci, [18] Cross-sectional study Moderate To assess the concentrations of HMGB-1, TNF-α, and IL-1β in the peri-implant crevicular fluid of CS and NS with and without peri-implantitis, as well as their clinical and radiographical peri-implant status.	15 CS 48.8 ± 7.8 y.o. 15M:0F N. of implants: 39	15 peri-implantitis Baseline (3.65 ± 0.96 y.): FMPS (%): 38.6 ± 14.2 BoP (%): 28.5 ± 7.8 PD: 5.8 ± 2.3 mm MBL: 2.8 ± 0.6 mm HMGB-1: 169 ± 85 pg/mL TNF-α: 342 ± 129 pg/mL IL-1β: 188 ± 32 pg/mL	PI was statistically significantly higher among CS with and without peri-implantitis and NS with peri-implantitis compared with NS in peri-implant health (*p* < 0.05). BoP, PD, and MBL were statistically significantly higher among CS and NS with peri-implantitis compared with CS and NS in peri-implant health (*p* < 0.05). HMGB-1 and IL-1β in peri-implant crevicular fluid were significantly higher in CS and NS with peri-implantitis compared with CS and NS in peri-implant health (*p* < 0.05). NS in peri-implant health reported the lowest levels of TNF-α compared with the other group (*p* < 0.05). A significant negative correlation of HMGB-1 was recorded for PI and MBL in NS in peri-implant health and CS with peri-implantitis, respectively (*p* < 0.05). A significant positive correlation between HMGB-1 and BoP in CS and NS with peri-implantitis (*p* < 0.005). A significant positive correlation between TNF-α and BoP in CS and NS with peri-implantitis (*p* < 0.05). A significant positive correlation between TNF-α and PD in CS with peri-implantitis (*p* < 0.05). A significant positive correlation of IL-1β was recorded for MBL in CS and NS with peri-implantitis, respectively (*p* < 0.05).	HMGB-1 in peri-implant crevicular fluid could represent a significant surrogate inflammatory marker of peri-implantitis.
	15 CS 46.2 ± 6.6 y.o. 15M:0F N. of implants: 28	15 peri-implant health Baseline (4.30 ± 1.40 y.): FMPS (%): 33.8 ± 10.6 BoP (%): 11.4 ± 6.4 PD: 1.4 ± 0.7 mm MBL: 1.5 ± 0.3 mm HMGB-1: 34 ± 18 pg/mL TNF-α: 119 ± 65 pg/mL IL-1β: 93 ± 37 pg/mL		
	15 NS 45.9 ± 10.2 y.o. 15M:0F N. of implants: 26	15 peri-implantitis Baseline (4.01 ± 0.82 y.): FMPS (%): 31.4 ± 15.8 BoP (%): 35.7 ± 14.6 PD: 5.2 ± 2.0 mm MBL: 2.6 ± 0.4 mm HMGB-1: 143 ± 68 pg/mL TNF-α: 289 ± 112 pg/mL IL-1β: 149 ± 76 pg/mL		
	15 NS 46.4 ± 7.2 y.o. 15M/0F N. of implants: 32	32 peri-implant health Baseline (45.6 ± 3.4 months): FMPS (%): 15.3 ± 6.1 BoP (%): 9.8 ± 4.5 PD: 1.1 ± 0.3 mm MBL: 0.6 ± 0.2 mm HMGB-1: 0.0 ± 0.0 pg/mL TNF-α: 22 ± 18 pg/mL IL-1β: 28 ± 13 pg/mL		
Al-Abdaly M.M.A.A., 2022 Int J Clin Med, [46] Cross-sectional study Serious No funding To assess the periodontal status and the salivary pH among E-Cigs, passive E-Cigs, and NS in relation to the duration of smoking.	30 NS 32.3 ± 14.07 y.o. 30M:0F N. of implants: MD	MD Periodontal status at baseline Plaque control record (%): 57.1 ± 23.5 BoP (%): 68.9 ± 23.01 PPD: 2.9 ± 0.8 mm CAL: 3.2 ± 1.5 mm MBL (%): 20.5 ± 10.6	Participants’ distribution in relation to periodontitis grading was statistically different between NS, E-Cigs, and passive E-Cigs (*p* < 0.05).	E-cigs and passive E-Cigs smoking have shown unwanted effects on periodontal status. E-cigs were associated with an increased periodontal disease grading. Passive E-Cigs with higher salivary pH values.
	30 E-Cigs 34.1 ± 8.84 y.o. 30M:0F N. of implants: MD	MD Periodontal status at baseline Plaque control record: 68.03 ± 27.23 BoP (%): 73.4 ± 21.6 PPD (%): 2.6 ± 1.1 mm CAL: 3.1 ± 2.2 mm MBL (%): 44.17 ± 19.1		
Ali D., 2022 BMC Oral Health, [19] Cross-sectional study Low No funding To assess the periodontal status and the AGEs in the gingival crevicular fluid among CS and WS.	28 CS 29.6 ± 1.2 y.o. 20M:8F N. of implants: MD	MD Periodontal status at baseline PI: 0.7 ± 0.005 GI: 0.6 ± 0.08 PPD: 0.7 ± 0.04 mm CAL: 0.4 ± 0.04 mm MBL (mesial): 0.8 ± 0.05 mm MBL (distal): 0.9 ± 0.005 mm Missing teeth: 5 AGEs: 225.4 ± 29.7 µg/mL	AGEs concentration was statistically significantly higher in CS and WS compared with NS (*p* < 0.001).	CS and WS with a short history of smoking showed similar clinical periodontal status compared with NS. However, AGEs concentration in the gingival crevicular fluid was lower in NS, potentially indicating an ongoing periodontal inflammatory process, yet not clinically manifested.
	28 WS 27.4 ± 0.7 y.o. 25M:3F N. of implants: MD	MD Periodontal status at baseline PI: 0.6 ± 0.07 GI: 0.7 ± 0.004 PPD: 0.8 ± 0.06 mm CAL: 0.5 ± 0.1 MBL (mesial): 1.01 ± 0.04 mm MBL (distal): 1.02 ± 0.08 mm Missing teeth: 6 AGEs: 216.6 ± 16.5 µg/mL		
	26 NS 26.8 ± 0.5 y.o. 21M:5F N. of implants: MD	MD Periodontal status at baseline PI: 0.6 ± 0.05 GI: 0.5 ± 0.005 PPD: 0.5 ± 0.1 mm CAL: 0.3 ± 0.003 MBL (mesial): 0.5 ± 0.04 mm MBL (distal): 0.4 ± 0.003 mm Missing teeth: 3 AGEs: 69.6 ± 3.2 µg/mL		
Ali D., 2022 BMC Oral Health, [20] Case-control study Low No funding To compare the periodontal status and the whole salivary IL-15 and IL-18 among CS, E-Cigs, and NS.	19 CS 52.6 ± 6.1 y.o. 15M:4F N. of implants: MD	MD Periodontal status at baseline PI: 3.1 ± 0.2 GI: 0.9 ± 0.04 PPD: 6.5 ± 0.2 mm CAL: 8.4 ± 0.5 mm MBL (mesial): 6.2 ± 0.7 mm MBL (distal): 6.3 ± 0.6 Missing teeth: 16.6 ± 2.3 Salivary IL-15: 189.5 ± 26.7 pg/mL Salivary IL-18: 2869.8 ± 285.6 pg/mL	PI, CAL, MBL (mesial and distal), and the number of missing teeth were statistically significantly higher in CS, E-Cigs, and NS with periodontitis compared with NS periodontally healthy (*p* < 0.001). GI was statistically significantly lower in CS, E-Cigs, and NS periodontally healthy compared with NS with periodontitis (*p* < 0.001). Salivary IL-15 and IL-18 were statistically significantly higher in CS and E-Cigs (*p* < 0.001). Salivary IL-15 and IL-18 were statistically significantly lower in NS periodontally healthy and NS with periodontitis (*p* < 0.001).	CS and NS showed similar clinical periodontal status. However, immunoinflammatory biomarkers (IL-15 and IL-19) in unstimulated whole saliva were lower in NS.
	18 E-Cigs 49.5 ± 2.3 y.o. 12M:6F N. of implants: MD	MD Periodontal status at baseline PI: 2.5 ± 0.2 GI: 1.05 ± 0.03 PPD: 5.7 ± 0.2 mm CAL: 7.1 ± 0.4 mm MBL (mesial): 5.8 ± 0.2 mm MBL (distal): 5.5 ± 0.2 mm Missing teeth: 10.6 ± 1.2 Salivary IL-15: 174.7 ± 19.2 pg/mL Salivary IL-18: 2793.1 ± 196.4 pg/mL		
	19 NS 50.7 ± 2.2 y.o. 13M:6F N. of implants: MD	19 periodontitis PI: 2.3 ± 0.2 GI: 3.3 ± 0.05 PPD: 6.1 ± 0.4 mm CAL: 7.8 ± 0.3 mm MBL (mesial): 5.7 ± 0.2 mm MBL (distal): 5.8 ± 0.3 mm Missing teeth: 12.2 ± 1.5 Salivary IL-15: 91.2 ± 14.3 pg/mL Salivary IL-18: 1537.9 ± 101.8 pg/mL		
	19 NS 48.1 ± 1.3 y.o. 14M:5F N. of implants: MD	19 periodontal health PI: 0.3 ± 0.05 GI: 0.5 ± 0.004 PPD: 1.2 ± 0.06 mm CAL: 0.2 ± 0.003 mm MBL (mesial): 0.4 ± 0.004 mm MBL (distal): 0.3 ± 0.004 mm Missing teeth: 2.2 ± 0.5 mm Salivary IL-15: 4.1 ± 0.2 pg/mL Salivary IL-18: 100.3 ± 2.5 pg/mL		
Ali D., 2023 Int Dent J, [21] Cross-sectional study Low Funding from Kuwait University To assess the peri-implant status and the AGEs in peri-implant sulcular fluid among CS and WS.	25 CS 51.3 ± 5.2 y.o. 25M:0F N. of implants: 25	MD Peri-implant status at baseline Baseline (7.1 ± 1.6 y. from implant function): mPI: 2.7 ± 0.4 mGI: 0.6 ± 0.004 PD: 4.7 ± 0.4 mm MBL (mesial): 2.7 ± 0.2 mm MBL (distal): 2.4 ± 0.3 mm AGEs: 611.1 ± 37.4 pg/mL	mPI, PD, and MBL (mesial and distal) were statistically significantly higher in CS and WS compared with NS (*p* < 0.01). AGEs concentration was statistically significantly higher in CS and WS compared with NS (*p* < 0.01).	WS and CS showed similar worsened clinical peri-implant status and higher AGEs in peri-implant sulcular fluid compared with NS.
	24 WS 53.8 ± 2.4 y.o. 24M:0F N. of implants: 24	MD Peri-implant status at baseline Baseline (8.0 ± 0.4 y. from implant function): mPI: 2.4 ± 0.2 mGI: 0.5 ± 0.007 PD: 4.5 ± 0.4 mm MBL (mesial): 2.5 ± 0.4 mm MBL (distal): 2.3 ± 0.2 mm AGEs: 587.8 ± 41.6 pg/mL		
	25 NS 49.6 ± 0.9 y.o. 25M:0F N. of implants: 25	MD Peri-implant status at baseline Baseline (7.2 ± 0.5 y. from implant function): mPI: 0.4 ± 0.07 mGI: 0.8 ± 0.1 PD: 1.7 ± 0.2 mm MBL (mesial): 0.3 ± 0.05 mm MBL (distal): 0.2 ± 0.004 mm AGEs: 79.5 ± 6.4 pg/mL		
Aljudabaidi S.M., 2025 Int Dent J, [22] Cross-sectional study Moderate Funding from the Princess Nourah bint Abdulrahman University, Riyadh, Saudi Arabia To assess the peri-implant clinical and radiographic status, and yeast species levels in the peri-implant crevicular fluid of CS and NS with and without peri-implantitis.	22 CS 58.4 ± 5.02 y.o. 22M:0F N. of implants: 22	22 peri-implantitis Baseline (5.6 ± 1.9 y. from implant function): mPI: 0.86 ± 0.07 mGI: 0.27 ± 0.16 PD: 5.9 ± 1.1 mm MBL (mesial): 5.2 ± 0.8 mm MBL (distal): 5.1 ± 0.6 mm	mPI, PD, and MBL (distal) were statistically significantly higher among CS and NS with peri-implantitis, compared with NS in peri-implant health (*p* < 0.01). mGI was statistically significantly higher among NS with peri-implantitis compared with CS with peri-implantitis and NS in peri-implant health (*p* < 0.01). PD was statistically correlated with oral yeast level in the peri-implant crevicular fluid in CS with peri-implantitis (*p* < 0.01). PD and mPI were statistically correlated with oral yeast level in the peri-implant crevicular fluid in CS with peri-implantitis (*p* < 0.01).	CS and NS with peri-implantitis showed similar peri-implant clinical and radiographic parameters. Oral yeast, particularly *Candida albicans,* seems to have a role in the progression of peri-implantitis.
	22 NS 56.5 ± 7.3 y.o. 22M:0F N. of implants: 22	22 peri-implantitis Baseline (5.1 ± 0.7 y. from implant function): mPI: 0.81 ± 0.08 mGI: 0.86 ± 0.1 PD: 5.3 ± 0.9 mm MBL (mesial): 4.92 ± 0.5 mm MBL (distal): 5.09 ± 0.6 mm		
	24 NS 54.8 ± 5.8 y.o. 24M:0F N. of implants: 24	24 peri-implant health Baseline (12.2 ± 2.3 y. from implant function): mPI: 0.23 ± 0.03 mGI: 0.2 ± 0.08 PD: 1.75 ± 0.07 mm MBL (mesial): 0.53 ± 0.08 mm MBL (distal): 0.6 ± 0.1 mm		
Alkan B., 2023 Medicine (Baltimore), [23] Cross-sectional study Serious No funding To assess the impact of heavy CS and generalized periodontitis on the histopathological status of the periodontium and local genotoxic damage of exfoliated oral epithelial cells.	20 CS 45.4 ± 8.85 y.o. 17M:3F N. of implants: MD	20 generalized periodontitis PI: 1.15 ± 0.32 BoP (%): 74.9 ± 11.9 PPD: 4.91 ± 0.6 mm CAL: 5.37 ± 0.8 mm	NS and CS in periodontal health showed statistically significantly lower PI, BoP, PPD, and CAL compared with CS and NS with generalized periodontitis (*p* < 0.01).	Heavy CS and generalized periodontitis can independently elicit histopathological and genotoxic damage, but their impact was exacerbated when combined.
	20 NS 50.1 ± 11.3 y.o. 9M:11F N. of implants: MD	20 generalized periodontitis PI: 1.01 ± 0.26 BoP (%): 87.85 ± 6.6 PPD: 4.64 ± 0.78 mm CAL: 4.92 ± 0.97 mm		
	20 CS 37.05 ± 10.47 y.o. 11M:9F N. of implants: MD	20 periodontal health PI: 0.43 ± 0.36 BoP (%): 1.72 ± 1.88 PPD: 2.04 ± 0.34 mm CAL: 2.15 ± 0.35 mm		
	20 NS 28.6 ± 8.02 y.o. 4M:16F N. of implants: MD	20 periodontal health PI: 0.51 ± 0.39 BoP (%): 2.32 ± 2.03 PPD: 2.29 ± 0.32 mm CAL: 2.38 ± 0.32 mm		
Almazyad R.K., 2024 J Pharm Bioall Sci, [24] Cross-sectional study Serious Funding from the Deanship of Graduate Studies and Scientific Research at Qassim University To compare the effects of E-Cigs and CS on periodontal health.	30 CS N/D y.o. N/D M:F N. of implants: MD	MD Periodontal status at baseline GI: 1.79 CAL: 3.44 mm Russell’s periodontal index: 5.92	No statistical difference	E-Cigs had similar clinical periodontal parameters compared to CS, but less pronounced, even if not statistically significant manner.
	30 E-Cigs N/D y.o. N/D M:F N. of implants: MD	MD Periodontal status at baseline GI: 1.21 CAL: 2.98 mm Russell’s periodontal index: 4.08		
Almeslet A.S., 2024 BMC Oral Health, [25] Cross-sectional study Moderate Funding from the Princess Nourah bint Abdulrahman University, Riyadh, Saudi Arabia To assess the peri-implant status and the oral yeast carriage among CS with peri-implant mucositis.	20 CS 50.1 ± 4.5 y.o. 20M:0F N. of implants: 20	20 peri-implant mucositis Baseline (3.15 ± 0.9 y. from implant function): mPI: 0.88 ± 0.05 mGI: 0.23 ± 0.03 PD: 5.8 ± 1.05 mm MBL (mesial): 0.54 ± 0.15 mm MBL (distal): 0.56 ± 0.17 mm	mPI and PD were significantly higher in CS with peri-implant mucositis compared with CS and NS in peri-implant health (*p* < 0.05). mPI and PD were significantly higher in NS with peri-implant mucositis compared with CS and NS in peri-implant health (*p* < 0.05). mGI was significantly higher in NS with peri-implant mucositis compared with CS with peri-implant mucositis and peri-implant health, and with NS in peri-implant health (*p* < 0.05).	Oral yeast carriage in subgingival biofilm and clinical parameters of peri-implant tissue were higher in moderate CS than in light CS with peri-implant mucositis compared with NS in peri-implant health.
	19 CS 52.6 ± 5.2 y.o. 19M:0F N. of implants: 19	19 peri-implant health Baseline (0.7 ± 0.5 y. from implant function): mPI: 0.2 ± 0.005 mGI: 0.23 ± 0.08 PD: 1.2 ± 0.16 mm MBL (mesial): 0.35 ± 0.12 mm MBL (distal): 0.38 ± 0.16 mm		
	21 NS 55.3 ± 5.8 y.o. 21M:0F N. of implants: 21	21 peri-implant mucositis Baseline (1.7 ± 0.8 y. from implant function): mPI: 0.54 ± 0.04 mGI: 0.84 ± 0.12 PD: 4.1 ± 0.08 mm MBL (mesial): 0.45 ± 0.16 mm MBL (distal): 0.48 ± 0.15 mm		
	20 NS 51.8 ± 5.1 y.o. 20M:0F N. of implants: 20	20 peri-implant health Baseline (5.2 ± 1.4 y. from implant function): mPI: 0.22 ± 0.06 mGI: 0.21 ± 0.08 PD: 1.1 ± 0.2 mm MBL (mesial): 0.26 ± 0.05 mm MBL (distal): 0.28 ± 0.07 mm		
Almeslet A.S., 2024 Oral Health Prev Dent, [26] Cross-sectional study Moderate Funding from the Princess Nourah bint Abdulrahman University, Riyadh, Saudi Arabia To assess the clinical and radiographical periodontal status and the levels of prostaglandin E2 in the whole saliva of CS and WS.	33 CS 47.5 ± 10.5 y.o. 25M:8F N. of implants: MD	MD Periodontal status at baseline PI: 0.83 ± 0.22 GI: 0.32 ± 0.25 PPD: 4.94 ± 0.95 mm CAL: 3.45 ± 0.97 mm MBL (mesial): 4.26 ± 0.72 mm MBL (distal): 4.31 ± 0.5 mm Missing teeth: 13.5 ± 2.5 Salivary prostaglandin E2: 231.5 ± 66.3 pg/mL	PI, GI, PD, MBL (mesial and distal), and the number of missing teeth were statistically significantly higher among CS and WS compared with NS (*p* < 0.05). Salivary prostaglandin E2 was statistically significantly higher among CS and WS compared with NS (*p* < 0.05).	CS and WS showed similar clinical and radiographical periodontal status, as well as levels of prostaglandin E2 in whole saliva. However, these parameters were statistically significantly worse both in CS and WS compare with NS.
	34 WS 45.4 ± 9.6 y.o. 30M:4F N. of implants: MD	MD Periodontal status at baseline PI: 0.78 ± 0.18 GI: 0.34 ± 0.27 PPD: 4.42 ± 0.81 mm CAL: 3.62 ± 1.2 mm MBL (mesial): 4.14 ± 1.1 mm MBL (distal): 4.21 ± 0.95 mm Missing teeth: 11.2 ± 1.3 Salivary prostaglandin E2: 243.1 ± 69.3 pg/mL		
	33 NS 45.1 ± 9.2 y.o. 30M:3F N. of implants: MD	MD Periodontal status at baseline PI: 0.11 ± 0.04 GI: 0.2 ± 0.1 PPD: 1.41 ± 0.7 mm CAL: 0 mm MBL (mesial): 0.9 ± 0.6 mm MBL (distal): 0.71 ± 0.4 mm Missing teeth: 2.8 ± 0.4 Salivary prostaglandin E2: 76.6 ± 10.6 pg/mL		
Almeslet A.S., 2025 Int Dent J, [27] Cross-sectional study Critical No funding To compare the oral yeast carriage among CS and NS with and without prediabetes.	47 CS 53.9 ± 3.7 y.o. 47M:0F N. of implants: MD	MD Periodontal status at baseline PI: 0.64 ± 0.07 GI: 0.41 ± 0.25 PPD: 4.44 ± 0.42 mm CAL: 2.84 ± 0.65 mm MBL (mesial): 4.64 ± 0.52 mm MBL (distal): 4.54 ± 0.42 mm Missing teeth: 7.59 ± 4.58	PI, PPD, and CAL were significantly higher in CS with or without prediabetes and in NS with prediabetes compared with NS without prediabetes (*p* < 0.05). GI was statistically significantly higher in NS with prediabetes compared with NS without prediabetes (*p* < 0.05). MBL (mesial and distal) was significantly higher in CS with prediabetes compared with NS with or without prediabetes (*p* < 0.05). The number of missing teeth was significantly higher in CS with prediabetes compared with CS without prediabetes and NS with or without prediabetes (*p* < 0.05).	In non-prediabetic CS, the severity of clinical periodontal status appears to determine the oral yeast carriage. In pre-diabetic CS, oral yeast carriage appears to be affected by hyperglycemia.
	45 NS 52.4 ± 3.9 y.o. 45M:0F N. of implants: MD	MD Periodontal status at baseline PI: 0.32 ± 0.15 GI: 0.52 ± 0.40 PPD: 2.77 ± 1.51 mm CAL: 1.22 ± 1.08 mm MBL (mesial): 1.79 ± 0.47 mm MBL (distal): 1.64 ± 0.38 mm Missing teeth: 2.05 ± 1.97		
Alnufaiy B., 2025 BMC Oral Health, [28] Cross-sectional study Low Funding from Prince Sattam bin Abdulaziz University To assess the impact of E-Cigs on periodontal health and proinflammatory cytokines in the whole saliva of males in Al-Khary city in Saudi Arabia.	25 E-Cigs 24.52 ± 2.29 y.o. 25M:0F N. of implants: MD	MD Periodontal status at baseline FMPS (%): 48.16 ± 20.70 BoP (%): 28.60 ± 13.74 PPD: 4.10 ± 1.87 mm CAL: 2.72 ± 0.89 mm MBL: 2.81 ± 0.53 mm Missing teeth ^‡^: 2.58 ± 2.91 Salivary IL-1β: 889.05 ± 540.56 pg/mL Salivary IL-6: 17.07 ± 8.21 pg/mL	CAL, PPD, and MBL in E-Cigs were statistically significantly higher compared with NS (*p* < 0.05). Salivary IL-1β was statistically significantly higher in E-Cigs compared with NS (*p* < 0.05) in bivariate analysis (t test). However, a nonsignificant difference was found in the multivariate model (*p* = 0.194).	E-Cigs had a negative impact on periodontal status (in particular on CAL, PPD, and MBL).
	28 NS 37 ± 1.4 y.o. 28 M:0F N. of implants: MD	MD Periodontal status at baseline FMPS (%): 41.00 ± 19.35 BoP (%): 34.03 ± 24.29 PPD: 2.72 ± 0.67 mm CAL: 1.64 ± 2.04 mm MBL: 1.59 ± 0.22 mm Missing teeth ^‡^: 1.6 ± 1.63 Salivary IL-1β: 640.75 ± 138.78 pg/mL Salivary IL-6: 19.49 ± 11.90 pg/mL		
Alqahtani A.S., 2022 Eur Rev Med Pharmacol Sci, [29] Cross-sectional study Moderate Funding from the Deanship of Scientific Research, Prince Sattam Bin Abdulaziz University Alkharj To compare periodontal treatment needs among CS, E-Cigs, and NS.	50 CS N/D y.o. 46M:4F N. of implants: MD	MD Periodontal status at baseline CPI: code 0 (n.0); code 1 (n.1); code 2 (n.9); code 3 (n.28); code 4 (n.12)	CPI showed a significant difference among CS, E-Cigs, and NS (*p* = 0.0001). Treatment needs based on the CPI showed a significant difference among CS, E-Cigs, and NS (*p* = 0.0001).	Smoking had an imperative role on the periodontal status regardless of the smoking types. E-Cigs registered less complicated treatment needs compared with CS.
	50 E-Cigs N/D y.o. 41M:9F N. of implants: MD	MD Periodontal status at baseline CPI: code 0 (n.1); code 1 (n.5); code 2 (n.28); code 3 (n.15); code 4 (n.1)		
	50 NS N/D y.o. 25M:25F N. of implants: MD	MD Periodontal status at baseline CPI: code 0 (n.9); code 1 (n.8); code 2 (n.19); code 3 (n.11); code 4 (n.3)		
Alshahrani A., 2023 Eur Rev Med Pharmacol Sci, [30] Cross-sectional study Low Funding from the King Saud University To assess the peri-implant clinical, radiographic, and prosthetic status in CS and NS with conventional or short implants, as well as the IL-1β and the matrix metalloproteinase-9 in the peri-implant crevicular fluid.	50 CS 54 ± 3.3 y.o. 50M:0F N. of implants: 62	MD peri-implant status Baseline (6.53 ± 0.91 y. from implant function): FMPS (%): 62.85 ± 6 BoP (%): 11.9 ± 7.85 MBL: 4.35 ± 0.25 mm IL-1β: 287.28 ± 20.55 pg/mL Matrix metalloproteinase-9: 109.93 ± 13.05 ng/mL	PI, MBL, and the percentage of PD ≥ 4 mm were statistically significantly higher in CS compared with NS (*p* < 0.01). BoP was statistically significantly higher in NS compared with CS (*p* < 0.01). Matrix metalloproteinase-9 and IL-1β levels in the peri-implant crevicular fluid were statistically significantly higher among CS compared with NS (*p* < 0.01).	PI, PD, BoP, MBL, and prosthetic parameters registered worsened values in CS compared with NS. Clinical, radiographical, and prosthetic parameters were comparable between conventional vs. short implants.
	50 NS 52.5 ± 3.65 y.o. 50M:0F N. of implants: 60	MD peri-implant status Baseline (6.18 ± 1.02 y. from implant function): FMPS (%): 28.65 ± 3.5 BoP (%): 37.45 ± 22.65 MBL: 2.2 ± 0.15 mm IL-1β: 124.93 ± 17.9 pg/mL Matrix metalloproteinase-9: 38.55 ± 17.3 ng/mL		
AlZamil A.F., 2023 Saudi Dent J, [31] Cross-sectional study Serious Funding from the Deanship of Scientific Research at King Saud University To evaluate the impact of smoking on the clinical periodontal parameters and the gingival crevicular fluid among CS and NS periodontally healthy subjects and those with periodontitis.	32 NS 33.4 ± 10.5 y.o. 18M:14F N. of implants: MD	32 periodontal health FMPS (%): 7.88 ± 1.60 GI (%): 0.00 ± 0.00 BoP (%): 8.69 ± 1.12 PPD: 2.72 ± 0.46 mm CAL: 0.00 ± 0.00 mm	Regardless of periodontal status, PI, GI, PPD, and CAL were statistically significantly lower in NS compared with CS (*p* < 0.05). Regardless of periodontal status, BoP was statistically significantly higher in NS compared with CS (*p* < 0.05). Regardless of smoking status, PI, PPD, CAL, and BoP were statistically significantly lower in periodontally healthy subjects compared with periodontitis subjects (*p* < 0.05).	CS was associated with higher PD, PI, CAL, and gingival crevicular fluid volume, but with lower GI and BoP than NS. The number of cigarettes smoked had an important role on gingival crevicular fluid volume, PI, GI, PPD, CAL, and BoP.
	32 CS 32.3 ± 6.3 y.o. 18M:14F N. of implants: MD	32 periodontal health FMPS (%): 19.75 ± 7.90 GI (%): 0.00 ± 0.00 BoP (%): 5.69 ± 1.38 PPD: 2.94 ± 0.56 mm CAL: 0.00 ± 0.00 mm		
	32 NS 50.1 ± 11.8 y.o. 18M:14F N. of implants: MD	32 periodontitis FMPS (%): 57.13 ± 9.98 GI (%): 2.50 ± 0.51 BoP (%): 60.19 ± 14.61 PPD: 5.69 ± 0.86 mm CAL: 2.13 ± 1.07 mm		
	64 CS 42.5 ± 10.6 y.o. 50M:14F N. of implants: MD	64 periodontitis FMPS (%): 53.69 ± 10.14 GI (%): 1.88 ± 0.57 BoP (%): 38.47 ± 12.72 PPD: 6.57 ± 1.47 mm CAL: 3.42 ± 1.51 mm		
Božac E., 2024 Clin Oral Invest, [32] Cross-sectional study Serious Funding from the Croatian Science Foundation To compare the clinical periodontal status and caries risk using the Cariogram among THS, CS, and NS.	23 HTPs N/D y.o. 3M:20F N. of implants: MD	MD Periodontal status at baseline PI: 0.48	CS had the higher FMPS, which was statistically significant compared with NS (*p* = 0.015), but not compared with HTPs.	HTPs and CS were related to clinical condition (including higher FMPS among CS), which affected caries activity, even if no significant difference was found in caries risk assessment.
	23 CS N/D y.o. 3M:20F N. of implants: MD	MD Periodontal status at baseline PI: 0.51		
	23 NS N/D y.o. 3M:20F N. of implants: MD	MD Periodontal status at baseline PI: 0.39		
Dewan H., 2023 Technol Health Care, [33] Cross-sectional study Moderate No funding To assess the levels of suPAR and TNF-α in the peri-implant sulcular fluid of CS and NS with peri-implantitis.	20 CS 55.2 ± 6.7 y.o. 20M:0F N. of implants: 20	20 peri-implantitis Baseline (8.2 ± 0.8 y. from implant function): mPI: 2.7 ± 0.3 mGI: 1.1 ± 0.08 PD: 5.2 ± 0.04 mm MBL (mesial): 5.5 ± 0.3 mm MBL (distal): 5.3 ± 0.4 mm suPAR: 5.35 ± 0.31 ng/mL TNF-α: 15.41 ± 2.32 ng/mL	mPI, PD, and MBL (mesial and distal) were statistically significantly higher among CS with peri-implantitis compared with NS with peri-implantitis or in peri-implant health (*p* < 0.01). mGI was statistically significantly higher among NS with peri-implantitis compared with CS with peri-implantitis and NS in peri-implant health (*p* < 0.01). suPAR and TNF-α in peri-implant crevicular fluid were significantly higher in CS and NS with peri-implantitis compared with NS in peri-implant health. In NS with peri-implantitis, the correlation between suPAR and TNF-α in peri-implant crevicular fluid and PD was statistically significant (*p* < 0.01).	suPAR and TNF-α were recorded in higher concentrations in the peri-implant sulcular fluid of CS and NS with peri-implantitis compared with NS in peri-implant health. In NS, suPAR and TNF-α were correlated with PD.
	20 NS 56.1 ± 3.5 y.o. 20M:0F N. of implants: 20	20 peri-implantitis Baseline (8.5 ± 0.3 y. from implant function): mPI: 2.1 ± 0.2 mGI: 2.6 ± 0.8 PD: 4.6 ± 0.1 mm MBL (mesial): 4.5 ± 0.2 mm MBL (distal): 4.2 ± 0.3 mm suPAR: 4.22 ± 0.16 ng/mL TNF-α: 10.62 ± 1.53 ng/mL		
	20 NS 55.1 ± 2.4 y.o. 20M:0F N. of implants: 20	20 peri-implant health Baseline (8.3 ± 0.2 y from implant function): mPI: 0.4 ± 0.05 mGI: 0.2 ± 0.005 PD: 0.6 ± 0.004 mm MBL (mesial): 0.3 ± 0.005 mm MBL (distal): 0.2 ± 0.002 mm suPAR: 0.25 ± 0.05 ng/mL TNF-α: 2.81 ± 0.37 ng/mL		
Gajendran *p*., 2022 J Int Oral Health, [34] Case-control cross-sectional study Low No funding To compare the levels of RANKL and cathepsin K in the gingival crevicular fluid among NS and CS with chronic periodontitis.	40 CS 41.42 ± 7.37 y.o. 40 M:0F N. of implants: MD	40 generalized chronic periodontitis PI: 1.67 ± 0.46 mSBI: 1.50 ± 0.37 PPD: 5.55 ± 0.33 mm CAL: 5.87 ± 0.42 mm RANKL: 1.95 ± 1.312 pg/mL Cathepsin K: 16.768 ± 12.40 pg/mL	PPD (*p* = 0.03) and CAL (*p* = 0.002) were significantly higher among CS compared with NS with generalized chronic periodontitis. mSBI was significantly higher among NS compared with CS with generalized chronic periodontitis (*p* < 0.012). Cathepsin K level in the gingival crevicular fluid was statistically significantly higher among CS compared with NS with generalized chronic periodontitis (*p* = 0.037). RANKL and cathepsin K showed statistically significant positive correlation in CS with generalized chronic periodontitis (*p* = 0.026).	RANKL level in the gingival crevicular fluid was not significantly modified by CS. Cathepsin K level was significantly higher in CS with generalized chronic periodontitis compared with NS with generalized chronic periodontitis. In CS, a positive correlation was found between RANKL and cathepsin K, suggesting that RANKL influences cathepsin K expression in CS.
	40 NS 41.33 ± 9.71 40M:0F N. of implants: MD	40 generalized chronic periodontitis PI: 1.77 ± 0.37 mSBI: 1.74 ± 0.47 PPD: 5.11 ± 0.84 mm CAL: 5.41 ± 0.77 mm RANKL: 1.67 ± 1.347 pg/mL Cathepsin K: 11.59 ± 8.15 pg/mL		
Hasan N.W.M., 2024 BMC Oral Health, [35] Cross-sectional study Low Funding from the Deanship of Scientific Research, Vice Presidency for Graduate Studies and Scientific Research, Universiti Kebangsaan Malaysia, Kuala Lumpur To assess the periodontal status, salivary pH, and cotinine concentrations among CS, E-Cigs and NS.	48 CS 34.83 ± 6.33 y.o. 48M:0F N. of implants: MD	MD Periodontal status at baseline FMPS (%): 47.63 ± 31.41 GI (%): 11.79 ± 5.96 PPD: 2.71 ± 0.60 mm CAL: 2.77 ± 0.64 mm	PPD and CAL were statistically significantly higher among CS compared with NS (*p* < 0.05). GI was statistically significantly higher among CS compared with NS and E-Cigs (*p* < 0.05).	CS was associated with worse periodontal status compared with E-Cigs and a lower pH of saliva.
	48 E-Cigs 31.77 ± 6.92 y.o. 48M:0F N. of implants: MD	MD Periodontal status at baseline FMPS (%): 36.79 ± 31.42 GI (%): 16.15 ± 8.94 PPD: 2.64 ± 0.64 mm CAL: 2.68 ± 0.66 mm		
	48 NS 34.48 ± 7.18 y.o. 48M:0F N. of implants: MD	MD Periodontal status at baseline FMPS (%): 36.40 ± 24.83 GI (%): 17.42 ± 17.12 PPD: 2.39 ± 0.58 mm CAL: 2.42 ± 0.60 mm		
Jabbar Najim R.A., 2025 F1000Res, [36] Case-control study Low No funding To assess the correlation between clinical parameters and NETosis biomarkers in whole saliva, as well as their potential indicators of inflammation in periodontitis stage III/IV in CS and NS.	30 CS 40.47 ± 11.27 y.o. 30M:0F N. of implants: MD	30 periodontitis (22 Stage III and 8 IV) FMPS (%): 60.84 ± 27.58 BoP (%): 34.29 ± 20.08 PPD: 4.777 ± 0.859 mm CAL: 5.695 ± 1.179 mm Salivary citrullinated histone H3: 2.95 ± 0.64 Salivary neutrophil elastase: 3.12 ± 0.81 Salivary calprotectin: 382.96 ± 88.50 Salivary myeloperoxidase: 369.06 ± 92.49	FMPS, BoP, and CAL were statistically significantly higher in CS and NS with periodontitis compared with NS in periodontal health (*p* < 0.05). CAL was statistically significantly higher in CS with periodontitis compared with NS with periodontitis. Salivary biomarkers (citrullinated histone H3, neutrophil elastase, calprotectin, myeloperoxidase) were significantly higher in both CS and NS periodontitis compared with NS in periodontal health (*p* < 0.001). CS with periodontitis exhibited significantly higher biomarker levels than NS with periodontitis (*p* < 0.05). A significant association was observed between BoP and calprotectin in CS with periodontitis. In NS with periodontitis, significant associations were found between BoP and neutrophil elastase and between CAL and myeloperoxidase (*p* < 0.05).	NETosis biomarkers in whole saliva showed good accuracy in distinguishing CS and NS with severe periodontitis compared with healthy controls. CS was associated with a higher risk of periodontitis.
	30 NS 43.90 ± 9.77 y.o. 3M:0F N. of implants: MD	30 periodontitis (20 stage III and 10 IV) FMPS (%): 54.11 ± 24.59 BoP (%): 42.29 ± 18.31 PPD: 4.50 ± 0.543 mm CAL: 5.045 ± 0.957 mm Salivary citrullinated histone H3: 2.24 ± 0.55 Salivary neutrophil elastase: 1.83 ± 0.45 Salivary calprotectin: 308.36 ± 117.23 Salivary myeloperoxidase: 302.67 ± 94.23		
	25 NS 36.72 ± 11.24 y.o. 25M:0F N. of implants: MD	25 periodontal health FMPS (%): 22.58 ± 10.03 BoP (%): 5.28 ± 2.41 Salivary citrullinated histone H3: 0.49 ± 0.59 Salivary neutrophil elastase: 0.63 ± 0.18 Salivary calprotectin: 153.12 ± 93.38 Salivary myeloperoxidase: 93.68 ± 22.0		
Li X., 2025 Oral Health Prev Dent, [37] Cross-sectional study Critical Funding from the Yantai Development Zone Science and Technology Leading Talents Project; Shandong Taishan Leading Talent Project; Technology-based small and medium enterprises innovation capacity enhancement project; Central leadership of local science and technology development; Qilu University of Science, education and industry integration innovation pilot project To compare the oral status and inflammatory factors in saliva among E-Cigs, CS, and NS male Chinese.	32 NS 20.94 ± 2.08 y.o. 32M:0F N. of implants: MD	32 periodontal health (inclusion criteria: BoP < 10%) PI: 1.41 PPD: 0.72 mm Salivary IL-8: 379.27 pg/mL Salivary IL-1β: 236.48 pg/mL Salivary IL-6: 5.95 pg/mL	PI was statistically significantly higher among CS compared with NS and E-Cigs (*p* < 0.05). PPD was statistically significantly higher among CS and E-Cigs compared with NS (*p* < 0.05). Salivary IL-8 in CS was statistically significantly higher compared with NS (*p* < 0.05) and E-Cigs (*p* < 0.001), and the content in E-Cigs was statistically lower compared with NS (*p* < 0.05). Salivary IL-1β in CS was statistically significantly higher compared with NS and E-Cigs (*p* < 0.05). Salivary IL-6 in E-Cigs was statistically significantly lower compared with CS and NS (*p* < 0.05).	E-Cigs registered a better oral status compared with CS. CS had statistically significantly higher levels of inflammatory cytokines compared with NS and E-Cigs, while E-Cigs had statistically significantly lower levels of IL-8 and IL-6 compared with CS.
	31 CS 20.71 ± 1.00 y.o. 31M:0F N. of implants: MD	32 periodontal health (inclusion criteria: BoP < 10%) PI: 2.76 PPD: 1.14 mm Salivary IL-8: 475.13 pg/mL Salivary IL-1β: 317.25 pg/mL Salivary IL-6: 5.68 pg/ml		
	20 E-Cigs 26.45 ± 3.72 y.o. 20M:0F N. of implants: MD	32 periodontal health (inclusion criteria: BoP < 10%) PI: 1.71 PPD: 1.135 mm Salivary IL-8: 260.17 pg/mL Salivary IL-1β: 184.16 pg/mL Salivary IL-6: 3.18 pg/mL		
Mayer Y., 2025 J Periodontol, [38] Prospective cohort study Low Funding from the Alpha-Bio Tec Company Ltd. To assess the effects of CS on implant survival rates and peri-implant MBL over 15 months.	11 CS 50.1 ± 12.1 y.o. 10M:1F N. of implants: 13	11 periodontal health Baseline (1.28 y. after implant placement): BoP (%): 57.1 PD: 3.11 ± 0.87 mm REC: 0 SUP (%): 18.2 MBL: 1.5 ± 0.3 mm	At 67 weeks from implant placement, CS showed statistically higher MBL compared with NS (*p* = 0.0008).	Heavy CS (≥20 cigarettes/day) significantly had higher MBL and lower implant survival rates.
	18 NS 53.0 ± 14.8 y.o. 10M:8F N. of implants: 18	18 periodontal health Baseline (1.28 y. after implant placement): BoP (%): 35.0 PD: 3.11 ± 0.81 mm REC: 0 SUP (%): 0 MBL: 0.7 ± 0.6 mm		
Mišković I., 2024 Dent J (Basel), [39] Cross-sectional study Low Funding from the Croatian Science Foundation To assess the association between CS, HTPs, and NS, and periodontal status.	22 NS 38.5 y.o. 8M:14F N. of implants: MD	MD Periodontal status at baseline FMPS (%): 57.85 FMBS (%): 57 PPD: 1.88 mm CAL: 2.27 mm REC: 0.31 mm Number of furcation involvement: 0 Tooth mobility: 0 Missing teeth: 1	PPD was statistically significantly higher in CS compared with HTPs (*p* = 0.042).	CS showed worse periodontal status compared with HTPs, which, however, had a negative effect on the periodontium.
	22 HTPs 37.0 y.o. 8M:14F N. of implants: MD	MD Periodontal status at baseline FMPS (%): 57.14 FMBS (%): 50.78 PPD: 2.51 mm CAL: 2.75 mm REC: 0.24 mm Number of furcation involvement: 0 Tooth mobility: 0 Missing teeth: 2		
	22 CS 38.0 y.o. 8M:14F N. of implants: MD	MD Periodontal status at baseline FMPS (%): 63.39 FMBS (%): 67.44 PPD: 3.42 mm CAL: 3.6 mm REC: 0.34 mm Number of furcation involvement: 0 Tooth mobility: 0 Missing teeth: 2		
Oyapero A., 2023 West Afr J Med, [40] Cross-sectional study Moderate No funding To assess the association between cardiovascular disease, dental caries, and periodontitis in Nigeria.	8 CS N/D y.o. N/D M:F N. of implants: MD	MD Periodontal status at baseline CAL: 5.13 ± 0.84 mm	CAL was significantly higher among subjects above 65 years, with high waist-hip ratio/obese/overweight, with high LDL, and with poor diastolic control, regardless of smoking status (*p* < 0.05).	Cardiovascular disease risk factors, including smoking, were associated with higher odds of increased CAL.
	260 NS N/D y.o. N/D M:F N. of implants: MD	MD Periodontal status at baseline CAL: 2.27 ± 0.76 mm		
Saxena V., 2025 Przegl Epidemiol, [41] Cross-sectional study Critical No funding To compare the subgingival microbial status among smokeless, CS, and NS.	52 NS 23.38 ± 0.97 y.o. 40M:12F N. of implants: MD	MD Periodontal status at baseline Approximate PI: 1.34 ± 0.18 BoP: 0.32 ± 0.001 PPD: 1.20 ± 0.17 mm	Approximate PI was significantly lower among NS compared with CS (*p* < 0.05).	A higher proportion of Gram-positive and Gram-negative cocci and bacilli, and coccobacilli was found among CS compared with NS.
	30 CS 31.67 ± 3.85 y.o. 26M:4F N. of implants: MD	MD Periodontal status at baseline Approximate PI: 1.63 ± 0.42 BoP: 0.11 ± 0.08 PPD: 1.24 ± 0.22 mm		
Shah R., 2024 J Oral Res Rev, [47] Cross-sectional study Serious No funding To compare the clinical periodontal status of CS and NS.	150 CS N/D y.o. N/D M:F N. of implants: MD	MD Periodontal status at baseline mPI: 1.48 ± 1.34 mGI: 0.78 ± 0.68 CAL: 2.92 ± 2.95	mPI, mGI, and CAL were significantly higher among CS compared with NS (*p* < 0.001).	CS harmed the clinical periodontal status. A longer history of CS had a higher severity effect compared with NS.
	150 NS N/D y.o. N/D M:F N. of implants: MD	MD Periodontal status at baseline mPI: 0.86 ± 0.86 mGI: 1.62 ± 1.52 CAL: 1.37 ± 1.08		
Singh D., 2024 J Pharm Bioall Sci, [42] Cross-sectional study Critical No funding To assess the clinical periodontal status, the blood levels of vitamin B12, erythrocyte sedimentation rate, and folic acid of CS and NS with type 2 diabetes mellitus and periodontitis.	60 CS N/D y.o. N/D M:F N. of implants: MD	60 chronic periodontitis PI: 1.67 ± 0.63 GI: 1.53 ± 0.79 BoP: 1.17 ± 0.56 PPD: 5.38 ± 0.36 mm CAL: 5.08 ± 1.37 mm	GI and PI were statistically significantly higher among NS with chronic periodontitis and type 2 diabetes mellitus compared with CS with chronic periodontitis. PPD was statistically significantly lower among CS with chronic periodontitis compared with NS with chronic periodontitis and type 2 diabetes mellitus, but was also statistically significantly higher compared with NS with chronic periodontitis and without diabetes.	CS and NS with periodontitis registered statistically lower periodontal disease severity, erythrocyte sedimentation rate, vitamin B12, and folic acid levels compared with NS with periodontitis but with type 2 diabetes mellitus.
	120 NS N/D y.o. N/D M:F N. of implants: MD	120 chronic periodontitis PI: 2.21 ± 0.73 GI: 2.15 ± 0.63 BoP: 1.97 ± 0.73 PPD: 5.55 ± 0.90 mm CAL: 4.83 ± 1.44 mm		
Soldati K.R., 2022 Braz Dent J, [43] Cross-sectional study Serious Funding from the National Council for Scientific and Technological Development and the Coordination for the Improvement of Higher Education Personnel Foundations To assess the levels of β-defensin 1 and 2 in the gingival crevicular fluid among CS and NS.	25 CS 43.88 ± 10.64 y.o. 12M:13F N. of implants: MD	25 periodontitis (Stage III/IV) FMPS (%): 35.01 ± 21.77 GI (%): 10.25 ± 15.03 BoP (%): 21.89 ± 23.72 PPD: 3.05 ± 0.72 mm β-defensin 1: N/D β-defensin 2: N/D	PI, BoP, PPD, and CAL were significantly lower in NS periodontally healthy compared with both CS and NS with periodontitis (*p* < 0.0001). GI was significantly lower in the NS periodontally healthy compared with CS with periodontitis (*p* = 0.0001). In healthy sites, β-defensin 1 was significantly higher in periodontally healthy NS than in patients with periodontitis, regardless of smoking status (*p* < 0.0001). In healthy sites, β-defensin 2 was significantly lower in NS with periodontitis compared with periodontally healthy NS (*p* = 0.0003). In periodontal sites of patients with periodontitis, CS had significantly higher β-defensin 2 and significantly lower β-defensin 1 than NS (*p* < 0.05). Within the NS with periodontitis, diseased sites showed significantly higher levels of both β-defensin 1 and 2 compared with healthy sites (*p* < 0.05). These differences were not retrieved within the CS with periodontitis.	In CS, the immune response may interfere with the regulation of β-defensin 1 and 2 in the gingival crevicular fluid. CS presented a lower concentration of β-defensin 1 and higher β-defensin 2 in diseased sites compared with NS, potentially indicating that the worsened severity of periodontitis in CS may be influenced by the altered expression of β-defensin.
	25 NS 40.32 ± 12 y.o. 9M:16F N. of implants: MD	25 periodontitis (Stage III/IV) FMPS (%): 29.62 ± 14.82 GI (%): 12.45 ± 7.72 BoP (%): 19.45 ± 14.73 PPD: 2.92 ± 0.54 mm β-defensin 1: N/D β-defensin 2: N/D		
	20 NS 25.79 ± 3.95 y.o. 5M:15F N. of implants: MD	20 periodontal health FMPS (%): 7.14 GI (%): 1.78 BoP (%): 2.98 PPD: 1.49 ± 0.29 mm β-defensin 1: N/D β-defensin 2: N/D		
Suman A., 2025 J Pharm Bioall Sci, [44] Cross-sectional study Moderate No funding To assess the pattern and degree of alveolar bone loss among NS and CS with chronic periodontitis using radiovisiography radiographs and transgingival probing.	25 CS N/D y.o. 25M:0F N. of implants: MD	25 chronic periodontitis PI: 2.90 ± 0.73 PPD: 4.99 ± 1.10 mm CAL: 4.81 ± 1.02 mm MBL (interproximal): 2.42 ± 0.91	PI, PPD, and CAL were statistically significantly higher in CS compared to NS (*p* < 0.05). Interproximal MBL was statistically significantly higher in the maxilla and incisors of the mandible of CS compared to NS (*p* < 0.05).	CS registered more bone loss than NS. Digital radiography had higher readability compared with transgingival probing in evaluating bone loss.
	25 NS N/D y.o. 25M:0F N. of implants: MD	25 chronic periodontitis PI: 1.62 ± 1.12 PPD: 3.56 ± 0.83 mm CAL: 3.25 ± 1.14 mm MBL (interproximal): 2.05 ± 0.74		
Tanik A., 2022 Am J Dent, [45] Retrospective study Moderate No funding To compare the effect of filtered vs. unfiltered CS on MBL around dental implants.	98 NS N/D y.o. N/D M:F N. of implants: N/D	MD Peri-implant status at baseline Baseline (4 y. after the implant placement): MBL (mesial): 1.12 ± 0.7 mm MBL (distal): 1.14 ± 0.7 mm	MBL significantly increased over time in NS, CS, and UCS (*p* < 0.001). At 4 years from implant placement, mean and mesial MBL were significantly higher in CS and UCS compared with NS (*p* < 0.001). Distal MBL also differed significantly among NS, CS, and UCS (*p* < 0.05), with significant differences between NS vs CS and NS vs UCS (*p* < 0.001).	Smoking was associated with increased MBL, and UCS exhibited greater bone loss than CS. Although a dose-response trend with daily cigarette consumption was observed, this association did not reach statistical significance.
	39 CS N/D y.o. N/D M:F N. of implants: N/D	MD Peri-implant status at baseline Baseline (4 y. after the implant placement): MBL (mesial): 1.58 ± 1.0 mm MBL (distal): 1.92 ± 1.0 mm		
	51 UCS N/D y.o. N/D M:F N. of implants: N/D	MD Peri-implant status at baseline Baseline (4 y. after the implant placement): MBL (mesial): 1.92 ± 0.9 mm MBL (distal): 2.01 ± 0.6 mm		
Taskaldiran E.S., 2024 Odontology [48] Cross-sectional study Moderate Funding from the Scientific Research Foundation of Gazi University To assess the concentration of IL-17 and IL-35 in unstimulated saliva and gingival crevicular fluid and the related impact on CS and NS with periodontitis.	19 CS 44.3 ± 9.6 y.o. 13M:6F N. of implants: MD	19 periodontitis PI: 2.54 ± 0.39 GI: 1.35 ± 0.27 BoP (%): 68.57 ± 19.57 PPD: 3.8 ± 0.58 mm CAL: 2.74 ± 1.52 mm IL-17: 131.37 ± 52.76 pg/mL IL-35: 4.24 ± 1.76 pg/µL Salivary IL-17: 83.08 ± 42.92 pg/mL Salivary IL-35: 3.25 ± 2.44 pg/µL	PI, GI, PPD, BoP, and CAL were statistically lower for NS in periodontal health compared with CS and NS with periodontitis (*p* < 0.05). GI was statistically lower in CS with periodontitis compared with NS with periodontitis (*p* < 0.05). IL-17 concentrations in the gingival crevicular fluid were statistically significantly lower in NS with periodontitis compared with CS with periodontitis and NS in periodontal health (*p* < 0.001). IL-35 concentrations in the gingival crevicular fluid were statistically significantly lower in NS in periodontal health compared with CS and NS with periodontitis (*p* < 0.001).	CS may affect the concentration of IL-17 and IL-35 in the saliva and gingival crevicular fluid, which may play a role in periodontal health and disease.
	20 NS 46 ± 9.74 y.o. 13M:7F N. of implants: MD	20 periodontitis PI: 2.32 ± 0.34 GI: 1.77 ± 0.19 BoP (%): 77.65 ± 19.63 PPD: 3.36 ± 0.4 mm CAL: 1.85 ± 1.01mm IL-17: 84.46 ± 28.92 pg/mL IL-35: 2.83 ± 0.96 pg/µL Salivary IL-17: 63.34 ± 24.43 pg/mL Salivary IL-35: 2.37 ± 1.17 pg/µL		
	18 NS 42.9 ± 12.8 y.o. 9M:9F N. of implants: MD	18 periodontal health PI: 0.47 ± 0.18 GI: 0.47 ± 0.17 BoP (%): 5.1 ± 1.96 PPD: 2.08 ± 0.19 mm CAL: 0.04 ± 0.18 mm IL-17: 136.94 ± 32.78 pg/mL IL-35: 5.23 ± 1.19 pg/µL Salivary IL-17: 59.39 ± 26.81 pg/mL Salivary IL-35: 1.93 ± 1.58 pg/µL		

Abbreviations: number, “n.”; years old, “y.o.”; years, “y.”; male, “M”; female, “F”; missing data, “MD”; not defined, “N/D”; *p* value, “*p*”; millimeters, “mm”; milliliters, “ml”; picogram, “pg”; nanogram, “ng”; microgram, “µg”; cigarette smokers or traditional combustible tobacco, “CS”; unfiltered cigarette smokers, “UCS”; heated tobacco products users, “HTPs”; electronic cigarette users, “E-Cigs”; waterpipe smokers, “WS”; non-smokers, “NS”; plaque index, “PI”; modified plaque index, “mPI”; full mouth plaque score, “FMPS”; gingival index, “GI”; modified gingival index, “mGI”; bleeding on probing, “BoP”; modified sulcular bleeding index, “mSBI”; suppuration, “SUP”; recession, “REC”; periodontal probing depth, “PPD”; probing depth (peri-implant), “PD”; clinical attachment level, “CAL”; community periodontal index, “CPI”; marginal bone level, “MBL”; interleukin, “IL”; tumor necrosis factor, TNF”; receptor activator of nuclear factor kappa-B ligand, “RANKL”; soluble urokinase plasminogen activator receptor, “suPAR”; high mobility group box chromosomal protein-1, “HMGB-1”; advanced glycation endproducts, “AGEs”. When studies reported the number of remaining teeth, the number of missing teeth was calculated assuming a full dentition of 32 teeth, as specified in the original study, “‡”.

**Table 3:** Demographic characteristics, periodontal clinical and radiographic parameters, and inflammatory biomarkers in gingival crevicular fluid and saliva among different users of inhaled tobacco and nicotine products. For each variable, the first reported value represents the overall mean of the investigated group, independent of periodontal status; stratified data based on periodontal status are shown when available. Data are expressed as sample-size-weighted means of the study-level means. The number of participants contributing to each outcome varies according to data availability across the included studies. Sample size (n.) refers to the number of subjects assessed for each specific parameter. Note: To enhance readability and presentation clarity, sub-categories or parameter rows with no available data across all included studies have been omitted from the table.

	NS	CS	HTPs	E-Cigs	WS
**Population**					
Sample size	1265	854	45	221	62
*NPS*	767	488	45	201	62
*Periodontal health*	167	83	-	20	-
*Periodontitis*	331	283	-	-	-
Mean age (y.o.)	37.99 ± 8.29 (n.637)	39.97 ± 11.61 (n.558)	37.00 (n.22)	32.49 ± 5.65 (n.141)	37.27 ± 9.01 (n.62)
*NPS*	36.15 ± 6.76 (n.284)	41.35 ± 11.64 (n.277)	37.00 (n.22)	33.49 ± 9.79 (n.121)	37.27 ± 9.01 (n.62)
*Periodontal health*	32.72 ± 8.30 (n.167)	29.12 ± 9.32 (n.83)	-	26.45 ± 3.72 (n.20)	-
*Periodontitis*	45.52 ± 10.18 (n.186)	42.61 ± 9.75 (n.198)	-	-	-
Gender ratio (M:F)	2.81:1 (n.731)	4.00:1 (n.437)	1:3.09 (n.45)	11.73:1 (n.191)	7.86:1 (n.62)
*NPS*	3.52:1 (n.357)	3.84:1 (n.300)	1:3.09 (n.45)	10.4:1 (n.191)	7.86:1 (n.62)
*Periodontal health*	1.77:1 (n.163)	2.61:1 (n.83)	-	20:0 (n.20)	-
*Periodontitis*	2.91:1 (n.211)	5.19:1 (n.223)	-	-	-
**Clinical parameters**					
CPI	1.82 (n.50)	3.02 (n.50)	-	2.2 (n.50)	-
*NPS*	1.82 (n.50)	3.02 (n.50)	-	2.2 (n.50)	-
*Periodontal health*	-	-	-	-	-
*Periodontitis*	-	-	-	-	-
Russell’s periodontal index	-	5.92 (n.30)	-	4.08 (n.30)	-
*NPS*	-	5.92 (n.30)	-	4.08 (n.30)	-
PI (mean score)	1.47 ± 0.88 (n.460)	1.51 ± 0.42 (n.365)	0.48 (n.23)	2.08 ± 0.20 (n.38)	0.70 ± 0.14 (n.62)
*NPS*	0.34 ± 0.20 (n.127)	0.99 ± 0.14 (n.150)	0.48 (n.23)	2.5 ± 0.2 (n.18)	0.70 ± 0.14 (n.62)
*Periodontal health*	0.86 ± 0.13 (n.69)	1.85 ± 0.36 (n.51)	-	1.71 (n.20)	-
*Periodontitis*	1.88 ± 0.64 (n.264)	1.89 ± 0.56 (n.164)	-	-	-
FMPS (%)	38.49 ± 18.50 (n.292)	47.31 ± 21.26 (n.221)	57.14 (n.22)	48.63 ± 27.90 (n.103)	-
*NPS*	45.92 ± 23.13 (n.128)	52.58 ± 31.41 (n.70)	57.14 (n.22)	48.63 ± 27.90 (n.103)	-
*Periodontal health*	12.45 ± 6.73 (n.77)	19.75 ± 7.90 (n.32)	-	-	-
*Periodontitis*	48.17 ± 17.36 (n.87)	51.55 ± 18.55 (n.119)	-	-	-
mPI (mean score)	0.86 ± 0.86 (n.150)	1.48 ± 1.34 (n.150)	-	-	-
*NPS*	0.86 ± 0.86 (n.150)	1.48 ± 1.34 (n.150)	-	-	-
Approximate PI (mean score)	1.34 ± 0.18 (n.52)	1.63 ± 0.42 (n.30)	-	-	-
*NPS*	1.34 ± 0.18 (n.52)	1.63 ± 0.42 (n.30)	-	-	-
GI (mean score)	1.36 ± 0.43 (n.364)	1.07 ± 0.49 (n.332)	-	1.15 ± 0.03 (n.48)	0.503 ± 0.200(n.62)
*NPS*	0.414 ± 0.27 (n.104)	0.748 ± 0.203 (n.157)	-	1.15 ± 0.03 (n.48)	0.503 ± 0.20 (n.62)
*Periodontal health*	0.260 ± 0.086 (n.69)	0 (n.32)	-	-	-
*Periodontitis*	2.28 ± 0.55 (n.191)	1.66 ± 0.65 (n.143)	-	-	-
GI (%)	12.71 ± 14.61 (n.93)	11.26 ± 9.99 (n.73)	-	16.15 ± 8.94 (n.48)	-
*NPS*	17.42 ± 17.12 (n.48)	11.79 ± 5.96 (n.48)	-	16.15 ± 8.94 (n.48)	-
*Periodontal health*	1.78 (n.20)	-	-	-	-
*Periodontitis*	12.45 ± 7.72 (n.25)	10.25 ± 15.03 (n.25)	-	-	-
mGI (mean score)	1.62 ± 1.52 (n.150)	0.78 ± 0.68 (n.150)	-	-	-
*NPS*	1.62 ± 1.52 (n.150)	0.78 ± 0.68 (n.150)	-	-	-
BoP (mean score)	1.47 ± 0.61 (n.172)	0.817 ± 0.46 (n.90)	-	-	-
*NPS*	0.31 ± 0.001 (n.52)	0.11 ± 0.08 (n.30)	-	-	-
*Periodontitis*	1.97 ± 0.73 (n.120)	1.17 ± 0.56 (n.60)	-	-	-
BoP (%)	37.8 ± 17.5 (n.322)	36.8 ± 14.9 (n.232)	50.78 (n.22)	53.0 ± 18.5 (n.55)	-
*NPS*	53.4 ± 23.6 (n.80)	4.16 ± 1.59 (n.52)	50.78 (n.22)	53.0 ± 18.5 (n.55)	-
*Periodontal health*	5.28 ± 1.88 (n.115)	67.44 (n.22)	-	-	-
*Periodontitis*	55.0 ± 15.6 (n.127)	43.3 ± 17.1 (n.158)	-	-	-
mSBI (mean score)	1.74 ± 0.47 (n.40)	1.50 ± 0.37 (n.40)	-	-	-
*Periodontitis*	1.74 ± 0.47 (n.40)	1.50 ± 0.37 (n.40)	-	-	-
PPD (mm)	3.22 ± 0.71 (n.756)	4.64 ± 0.80 (n.321)	2.51 (n.22)	3.07 ± 1.09 (n.141)	2.78 ± 0.60 (n.62)
*NPS*	2.23 ± 0.78 (n.314)	3.53 ± 0.58 (n.276)	2.51 (n.22)	3.39 ± 1.09 (n.121)	2.78 ± 0.60 (n.62)
*Periodontal health*	1.74 ± 0.32 (n.141)	2.05 ± 0.49 (n.83)	-	1.135 (n.20)	-
*Periodontitis*	4.95 ± 0.80 (n.301)	5.41 ± 0.93 (n.234)	-	-	-
CAL (mm)	2.93 ± 1.04 (n.1037)	3.55 ± 1.77 (n.695)	2.75 (n.22)	3.36 ± 1.25 (n.151)	2.21 ± 0.89 (n.62)
*NPS*	1.82 ± 0.99 (n.642)	3.15 ± 2.05 (n.385)	2.75 (n.22)	3.36 ± 1.25 (n.151)	2.21 ± 0.89 (n.62)
*Periodontal health*	0.59 ± 0.22 (n.89)	0.83 ± 0.35 (n.52)	-	-	-
*Periodontitis*	4.51 ± 1.16 (n.306)	4.68 ± 1.23 (n.258)	-	-	-
REC (mm)	0.31 (n.22)	0.34 (n.22)	0.24 (n.22)	-	-
*NPS*	0.31 (n.22)	0.34 (n.22)	0.24 (n.22)	-	-
N. of missing teeth	3.14 ± 1.45 (n.144)	8.74 ± 3.62 (n.149)	2 (n.22)	5.94 ± 2.36 (n.43)	8.85 ± 1.30 (n.62)
*NPS*	2.13 ± 1.52 (n.106)	8.74 ± 3.62 (n.149)	2 (n.22)	5.94 ± 2.36 (n.43)	8.85 ± 1.30 (n.62)
*Periodontal health*	2.2 ± 0.5 (n.19)	-	-	-	-
*Periodontitis*	12.2 ± 1.5 (n.19)	-	-	-	-
N. of furcation involvements	0 (n.22)	0 (n.22)	0 (n.22)	-	-
*NPS*	0 (n.22)	0 (n.22)	0 (n.22)	-	-
Tooth mobility	0 (n.22)	0 (n.22)	0 (n.22)	-	-
*NPS*	0 (n.22)	0 (n.22)	0 (n.22)	-	-
**Radiographical parameters**					
MBL mesial (mm)	1.68 ± 0.40 (n.142)	3.93 ± 0.56 (n.127)	-	5.8 ± 0.2 (n.18)	2.73 ± 0.82 (n.62)
*NPS*	1.19 ± 0.46 (n.104)	3.93 ± 0.56 (n.127)	-	5.8 ± 0.2 (n.18)	2.73 ± 0.82 (n.62)
*Periodontal health*	0.4 ± 0.004 (n.19)	-	-	-	-
*Periodontitis*	5.7 ± 0.2 (n.19)	-	-	-	-
MBL distal (mm)	1.58 ± 0.31 (n.142)	3.94 ± 0.43 (n.127)	-	5.5 ± 0.2 (n.18)	2.77 ± 0.71 (n.62)
*NPS*	1.04 ± 0.34 (n.104)	3.94 ± 0.43 (n.127)	-	5.5 ± 0.2 (n.18)	2.77 ± 0.71 (n.62)
*Periodontal health*	0.3 ± 0.004 (n.19)	-	-	-	-
*Periodontitis*	5.8 ± 0.3 (n.19)	-	-	-	-
MBL mean (mm)	1.98 ± 0.64 (n.53)	2.05 ± 0.74 (n.25)	-	2.81 ± 0.53 (n.25)	-
*NPS*	1.59 ± 0.22 (n.28)	-	-	2.81 ± 0.53 (n.25)	-
*Periodontal health*	-	-	-	-	-
*Periodontitis*	2.42 ± 0.91 (n.25)	2.05 ± 0.74 (n.25)	-	-	-
MBL (%)	20.5 ± 10.6 (n.30)	-	-	44.17 ± 19.1 (n.30)	-
*NPS*	20.5 ± 10.6 (n.30)	-	-	44.17 ± 19.1 (n.30)	-
**Inflammatory biomarkers** Gingival crevicular fluid					
IL-35 (pg/µL)	3.97 ± 1.08 (n.38)	4.24 ± 1.76 (n.19)	-	-	-
*Periodontal health*	5.23 ± 1.19 (n.18)	-	-	-	-
*Periodontitis*	2.83 ± 0.96 (n.20)	4.24 ± 1.76 (n.19)	-	-	-
IL-17 (pg/mL)	109.32 ± 30.81 (n.38)	131.37 ± 52.76 (n.19)	-	-	-
*Periodontal health*	136.94 ± 32.78 (n.18)	-	-	-	-
*Periodontitis*	84.46 ± 28.92 (n.20)	131.37 ± 52.76 (n.19)	-	-	-
AGEs (µg/mL)	69.6 ± 3.2 (n.26)	225.4 ± 29.7 (n.28)	-	-	216.6 ± 16.5 (n.28)
*NPS*	69.6 ± 3.2 (n.26)	225.4 ± 29.7 (n.28)	-	-	216.6 ± 16.5 (n.28)
RANKL (pg/mL)	1.67 ± 1.347 (n.40)	1.95 ± 1.312 (n.40)	-	-	-
*Periodontitis*	1.67 ± 1.347 (n.40)	1.95 ± 1.312 (n.40)	-	-	-
Cathepsin K (pg/mL)	11.59 ± 8.15 (n.40)	16.768 ± 12.40 (n.40)	-	-	-
*Periodontitis*	11.59 ± 8.15 (n.40)	16.768 ± 12.40 (n.40)	-	-	-
Saliva					
IL-35 (pg/µL)	2.16 ± 1.38 (n.38)	3.25 ± 2.44 (n.19)	-	-	-
*Periodontal health*	1.93 ± 1.58 (n.18)	-	-	-	-
*Periodontitis*	2.37 ± 1.17 (n.20)	3.25 ± 2.44 (n.19)	-	-	-
IL-18 (pg/mL)	819.1 ± 72.0 (n.38)	2869.8 ± 285.6 (n.19)	-	2793.1 ± 196.4 (n.18)	-
*NPS*	819.1 ± 72.0 (n.38)	2869.8 ± 285.6 (n.19)	-	2793.1 ± 196.4 (n.18)	-
IL-17 (pg/mL)	61.47 ± 25.57 (n.38)	83.08 ± 42.92 (n.19)	-	-	-
*Periodontal health*	59.39 ± 26.81 (n.18)	-	-	-	-
*Periodontitis*	63.34 ± 24.43 (n.20)	83.08 ± 42.92 (n.19)	-	-	-
IL-15 (pg/mL)	47.65 ± 10.11 (n.38)	189.5 ± 26.7 (n.19)	-	174.7 ± 19.2 (n.18)	-
*NPS*	47.65 ± 10.11 (n.38)	189.5 ± 26.7 (n.19)	-	174.7 ± 19.2 (n.18)	-
IL-8 (pg/mL)	379.27 (n.32)	475.13 (n.31)	-	260.17 (n.20)	-
*NPS*	-	-	-	-	-
*Periodontal health*	379.27 (n.32)	475.13 (n.31)	-	260.17 (n.20)	-
IL-6 (pg/mL)	12.27 ± 11.90 (n.60)	5.68 (n.31)	-	10.90 ± 8.21 (n.45)	-
*NPS*	19.49 ± 11.90 (n.28)	-	-	17.07 ± 8.21 (n.25)	-
*Periodontal health*	5.95 (n.32)	5.68 (n.31)	-	3.18 (n.20)	-
IL-1β (pg/mL)	425.14 ± 138.78 (n.60)	317.25 (n.31)	-	576.88 ± 540.56 (n.45)	-
*NPS*	640.75 ± 138.78 (n.28)	-	-	889.05 ± 540.56 (n.25)	-
*Periodontal health*	236.48 (n.32)	317.25 (n.31)	-	184.16 (n.20)	-
Prostaglandin E2 (pg/mL)	76.6 ± 10.6 (n.33)	231.5 ± 66.3 (n.33)	-	-	243.1 ± 69.3 (n.34)
*NPS*	76.6 ± 10.6 (n.33)	231.5 ± 66.3 (n.33)	-	-	243.1 ± 69.3 (n.34)
Citrullinated histone H3	1.45 ± 0.57 (n.55)	2.95 ± 0.64 (n.30)	-	-	-
*Periodontal health*	0.49 ± 0.59 (n.25)	2.95 ± 0.64 (n.30)	-	-	-
*Periodontitis*	2.24 ± 0.55 (n.30)	-	-	-	-
Neutrophil elastase	1.28 ± 0.35 (n.55)	3.12 ± 0.81 (n.30)	-	-	-
*Periodontal health*	0.63 ± 0.18 (n.25)	3.12 ± 0.81 (n.30)	-	-	-
*Periodontitis*	1.83 ± 0.45 (n.30)	-	-	-	-
Calprotectin	238.5 ± 107.0 (n.55)	382.96 ± 88.50 (n.30)	-	-	-
*Periodontal health*	153.12 ± 93.38 (n.25)	382.96 ± 88.50 (n.30)	-	-	-
*Periodontitis*	308.36 ± 117.23 (n.30)	-	-	-	-
Myeloperoxidase	206.76 ± 71.3 (n.55)	369.06 ± 92.49 (n.30)	-	-	-
*Periodontal health*	93.68 ± 22.0 (n.25)	369.06 ± 92.49 (n.30)	-	-	-
*Periodontitis*	302.67 ± 94.23 (n.30)	-	-	-	-

Abbreviations: number, “n.”; years old, “y.o.”; male, “M”; female, “F”; not defined, “N/D”; percentage, “%”; millimeters, “mm”; milliliters, “ml”; picogram, “pg”; microgram, “µg”; non-stratified periodontal status, “NPS”; cigarette smokers or traditional combustible tobacco, “CS”; heated tobacco products users, “HTPs”; electronic cigarette users, “E-Cigs”; waterpipe smokers, “WS”; non-smokers, “NS”; community periodontal index, “CPI”; plaque index, “PI; full mouth plaque score, “FMPS”; modified plaque index, “mPI”; gingival index, “GI”; modified gingival index, “mGI”; bleeding on probing, “BoP”; modified sulcular bleeding index, “mSBI”; periodontal probing depth, “PPD”; clinical attachment level, “CAL”; recession, “REC”; marginal bone level, “MBL”; interleukin, “IL”; receptor activator of nuclear factor kappa-B ligand, “RANKL”; advanced glycation endproducts, “AGEs”.

**Table 4:** Demographic characteristics, peri-implant clinical and radiographic parameters, and inflammatory biomarkers in peri-implant crevicular fluid among different users of inhaled tobacco and nicotine products. For each variable, the first reported value represents the overall mean of the investigated group, independent of periodontal status; stratified data based on peri-implant status are shown when available. Data are expressed as sample-size-weighted means of the study-level means. The number of participants contributing to each outcome varies according to data availability across the included studies. Sample size (n.) refers to the number of subjects assessed for each specific parameter. Note: To enhance readability and presentation clarity, sub-categories or parameter rows with no available data across all included studies have been omitted from the table.

	NS	CS	UCS	WS
**Population**				
Sample size	348	236	51	24
*NpS*	191	125	51	24
*Peri-implant health*	79	34	-	-
*Peri-implant mucositis*	21	20	-	-
*Peri-implantitis*	57	57	-	-
N. of implants	288 (n.250)	220 (n.197)	N/D (n.51)	24 (n.24)
*NpS*	103 (n.93)	100 (n.86)	N/D (n.51)	24 (n.24)
*Peri-implant health*	96 (n.79)	19 (n.34)	-	-
*Peri-implant mucositis*	21 (n.21)	20 (n.20)	-	-
*Peri-implantitis*	68 (n.57)	81 (n.57)	-	-
Mean age (y.o.)	53.63 ± 5.48 (n.250)	52.55 ± 5.82 (n.197)	N/D (n.51)	53.8 ± 2.4 (n.24)
*NpS*	51.82 ± 6.99 (n.93)	52.72 ± 5.65 (n.86)	N/D (n.51)	53.8 ± 2.4 (n.24)
*Peri-implant health*	52.52 ± 5.30 (n.79)	49.78 ± 5.86 (n.34)	-	-
*Peri-implant mucositis*	55.3 ± 5.8 (n.21)	50.1 ± 4.5 (n.20)	-	-
*Peri-implantitis*	53.57 ± 7.21 (n.57)	49.84 ± 7.81 (n.57)	-	-
Gender ratio (M:F)	242:8 (n.250)	196:1 (n.197)	N/D (n.51)	24:0 (n.24)
*NpS*	85:8 (n.93)	85:1 (n.86)	N/D (n.51)	24:0 (n.24)
*Peri-implant health*	79:0 (n.79)	34:0 (n.34)	-	-
*Peri-implant mucositis*	21:0 (n.21)	20:0 (n.20)	-	-
*Peri-implantitis*	57:0 (n.57)	57:0 (n.57)	-	-
**Clinical parameters**				
mPI (mean score)	0.645 ± 0.60 (n.152)	1.53 ± 1.06 (n.106)	-	2.4 ± 0.2 (n.24)
*NpS*	0.4 ± 0.07 (n.25)	2.7 ± 0.4 (n.25)	-	2.4 ± 0.2 (n.24)
*Peri-implant health*	0.28 ± 0.095 (n.64)	0.2 ± 0.005 (n.19)	-	-
*Peri-implant mucositis*	0.54 ± 0.04 (n.21)	0.88 ± 0.05 (n.20)	-	-
*Peri-implantitis*	1.43 ± 0.66 (n.42)	1.74 ± 0.94 (n.42)	-	-
FMPS (%)	24.66 ± 10.07 (n.97)	52.86 ± 15.81 (n.80)	-	-
*NpS*	28.65 ± 3.5 (n.50)	62.85 ± 6 (n.50)	-	-
*Peri-implant health*	15.3 ± 6.1 (n.32)	33.8 ± 10.6 (n.15)	-	-
*Peri-implantitis*	31.4 ± 15.8 (n.15)	38.6 ± 14.2 (n.15)	-	-
mGI (mean score)	0.80 ± 0.82 (n.152)	0.49 ± 0.34 (n.106)	-	0.5 ± 0.007 (n.24)
*NpS*	0.8 ± 0.1 (n.25)	0.6 ± 0.004 (n.25)	-	0.5 ± 0.007 (n.24)
*Peri-implant health*	0.203 ± 0.067 (n.64)	0.23 ± 0.08 (n.19)	-	-
*Peri-implant mucositis*	0.84 ± 0.12 (n.21)	0.23 ± 0.03 (n.20)	-	-
*Peri-implantitis*	1.69 ± 1.03 (n.42)	0.67 ± 0.43 (n.42)	-	-
BoP (%)	29.14 ± 21.7 (n.115)	20.01 ± 11.23 (n.91)	-	-
*NpS*	36.8 ± 22.65 (n.68)	20.05 ± 7.85 (n.61)	-	-
*Peri-implant health*	9.8 ± 4.5 (n.32)	11.4 ± 6.4 (n.15)	-	-
*Peri-implantitis*	35.7 ± 14.6 (n.15)	28.5 ± 7.8 (n.15)	-	-
SUP (%)	0 (n.18)	18.2 (n.11)	-	-
*NpS*	0 (n.18)	18.2 (n.11)	-	-
PD (mm)	2.68 ± 0.41 (n.217)	4.30 ± 0.77 (n.147)	-	4.5 ± 0.4 (n.24)
*NpS*	2.29 ± 0.54 (n.43)	4.21 ± 0.58 (n.36)	-	4.5 ± 0.4 (n.24)
*Peri-implant health*	1.16 ± 0.20 (n.96)	1.29 ± 0.48 (n.34)	-	-
*Peri-implant mucositis*	4.1 ± 0.08 (n.21)	5.8 ± 1.05 (n.20)	-	-
*Peri-implantitis*	5.03 ± 0.91 (n.57)	5.63 ± 1.04 (n.57)	-	-
REC (mm)	0 (n.18)	0 (n.11)	-	-
*NpS*	0 (n.18)	0 (n.11)	-	-
**Radiographical parameters**				
MBL (mesial)	1.39 ± 0.36 (n.250)	2.56 ± 0.50 (n.145)	1.92 ± 0.9 (n.51)	2.5 ± 0.4 (n.24)
*NpS*	0.95 ± 0.57 (n.123)	2.02 ± 0.69 (n.64)	1.92 ± 0.9 (n.51)	2.5 ± 0.4 (n.24)
*Peri-implant health*	0.37 ± 0.047 (n.64)	0.35 ± 0.12 (n.19)	-	-
*Peri-implant mucositis*	0.45 ± 0.16 (n.21)	0.54 ± 0.15 (n.20)	-	-
*Peri-implantitis*	4.71 ± 0.36 (n.42)	5.34 ± 0.56 (n.42)	-	-
MBL (distal)	1.46 ± 0.38 (n.250)	2.59 ± 0.51 (n.145)	2.01 ± 0.6 (n.51)	2.3 ± 0.2 (n.24)
*NpS*	0.95 ± 0.56 (n.123)	2.11 ± 0.73 (n.64)	2.01 ± 0.6 (n.51)	2.3 ± 0.2 (n.24)
*Peri-implant health*	0.38 ± 0.06 (n.64)	0.38 ± 0.16 (n.19)	-	-
*Peri-implant mucositis*	0.48 ± 0.15 (n.21)	0.56 ± 0.17 (n.20)	-	-
*Peri-implantitis*	5.09 ± 0.46 (n.42)	5.29 ± 0.50 (n.42)	-	-
MBL mean (mm)	1.57 ± 0.27 (n.115)	3.28 ± 0.32 (n.91)	-	-
*NpS*	1.80 ± 0.33 (n.68)	3.84 ± 0.26 (n.61)	-	-
*Peri-implant health*	0.6 ± 0.2 (n.32)	1.5 ± 0.3 (n.15)	-	-
*Peri-implantitis*	2.6 ± 0.4 (n.15)	2.8 ± 0.6 (n.15)	-	-
**Inflammatory biomarkers** Peri-implant crevicular fluid				
HMGB-1 (pg/mL)	45.64 ± 21.7 (n.47)	101.5 ± 51.5 (n.30)	-	-
*Peri-implant health*	0.0 ± 0.0 (n.32)	34 ± 18 (n.15)	-	-
*Peri-implantitis*	143 ± 68 (n.15)	169 ± 85 (n.15)	-	-
AGEs (µg/mL)	79.5 ± 6.4 (n.25)	611.1 ± 37.4 (n.25)	-	587.8 ± 41.6 (n.24)
*NpS*	79.5 ± 6.4 (n.25)	611.1 ± 37.4 (n.25)	-	587.8 ± 41.6 (n.24)
IL-1β (pg/mL)	96.7 ± 25.3 (n.97)	232.24 ± 25.78 (n.80)	-	-
*NpS*	124.93 ± 17.9 (n.50)	287.28 ± 20.55 (n.50)	-	-
*Peri-implant health*	28 ± 13 (n.32)	93 ± 37 (n.15)	-	-
*Peri-implantitis*	149 ± 76 (n.15)	188 ± 32 (n.15)	-	-
TNF-α (pg/mL)	61.0 ± 26.36 (n.87)	144.46 ± 59.13 (n.50)	-	-
*Peri-implant health*	14.62 ± 11.22 (n.52)	119 ± 65 (n.15)	-	-
*Peri-implantitis*	129.92 ± 48.87 (n.35)	155.38 ± 56.61 (n.35)	-	-
suPAR (pg/mL)	2.24 ± 0.105 (n.40)	5.35 ± 0.31 (n.20)	-	-
*Peri-implant health*	0.25 ± 0.05 (n.20)	-	-	-
*Peri-implantitis*	4.22 ± 0.16 (n.20)	5.35 ± 0.31 (n.20)	-	-
Matrix metalloproteinase-9 (ng/mL)	38.55 ± 17.3 (n.50)	109.93 ± 13.05 (n.50)	-	-
*NpS*	38.55 ± 17.3 (n.50)	109.93 ± 13.05 (n.50)	-	-

Abbreviations: number, “n.”; years old, “y.o.”; male, “M”; female, “F”; not defined, “N/D”; millimeters, “mm”; milliliters, “mL”; picogram, “pg”; nanogram, “ng”; non-stratified peri-implant status, “NpS”; cigarette smokers or traditional combustible tobacco, “CS”; unfiltered cigarette smokers, “UCS”; waterpipe smokers, “WS”; non-smokers, “NS”; modified plaque index, “mPI”; full mouth plaque score, “FMPS”; modified gingival index, “mGI”; bleeding on probing, “BoP”; suppuration, “SUP”; recession, “REC”; probing depth (peri-implant), “PD”; marginal bone level, “MBL”; interleukin, “IL”; tumor necrosis factor, TNF”; soluble urokinase plasminogen activator receptor, “suPAR”; high mobility group box chromosomal protein-1, “HMGB-1”; advanced glycation endproducts, “AGEs”.

## Data Availability

No new data were created or analyzed in this study. Data sharing is not applicable to this article.

## Supplementary Materials

The following supporting information can be downloaded at https://www.mdpi.com/article/10.3390/dj14090562/s1, Supplementary File S1: PRISMA 2020 checklist; Supplementary File S2: Risk of bias.

## Appendix A

### Advanced Search Strategy

**Database** **Date Restrictions** **Filters**	**Advanced Search Strategy**
PubMed/MEDLINE From 25 April 2022 to 23 December 2025 English language	((“Periodontal disease”[All Fields] OR (“periodontal”[All Fields] OR “periodontally”[All Fields] OR “periodontically”[All Fields] OR “periodontics”[MeSH Terms] OR “periodontics”[All Fields] OR “periodontic”[All Fields] OR “periodontitis”[MeSH Terms] OR “periodontitis”[All Fields] OR “periodontitides”[All Fields]) OR “peri-implant disease”[All Fields] OR “peri-implantitis”[All Fields] OR “dental implant”[All Fields] OR “implant loss”[All Fields] OR “plaque index”[All Fields] OR “gingival index”[All Fields] OR “bleeding on probing”[All Fields] OR “probing depth”[All Fields] OR “tooth loss”[All Fields] OR “missing teeth”[All Fields] OR “marginal bone level”[All Fields] OR “IL-1b”[All Fields] OR “IL-8”[All Fields] OR “IL-6”[All Fields] OR “TNF-a”[All Fields] OR “MMP-1”[All Fields] OR “MMP-8”[All Fields] OR “IFN-y”[All Fields] OR “IL-4”[All Fields] OR “IL-9”[All Fields] OR “IL-10”[All Fields] OR “IL-13”[All Fields] OR “OPG”[All Fields] OR “RANK-L”[All Fields]) AND (“cigarett”[All Fields] OR “cigarette s”[All Fields] OR “cigaretts”[All Fields] OR “tobacco products”[MeSH Terms] OR (“tobacco”[All Fields] AND “products”[All Fields]) OR “tobacco products”[All Fields] OR “cigarette”[All Fields] OR “cigarettes”[All Fields] OR “e-cigarette”[All Fields] OR “vaping cigarette”[All Fields] OR “electronic cigarette”[All Fields] OR “electronic nicotine delivery system”[All Fields] OR “Heat-Not-Burn Tobacco”[All Fields] OR (“vaping”[MeSH Terms] OR “vaping”[All Fields] OR “vape”[All Fields] OR “electronic nicotine delivery systems”[MeSH Terms] OR (“electronic”[All Fields] AND “nicotine”[All Fields] AND “delivery”[All Fields] AND “systems”[All Fields]) OR “electronic nicotine delivery systems”[All Fields]) OR (“vaped”[All Fields] OR “vaping”[MeSH Terms] OR “vaping”[All Fields] OR “vapes”[All Fields]))) AND ((2022/4/25:2025/12/23[pdat]) AND (english[Filter]))
Scopus From 2022 to 23 December 2025 English language	TITLE-ABS-KEY (“Periodontal disease” OR periodontitis OR “peri-implant disease” OR “peri-implantitis” OR “dental implant” OR “implant loss” OR “plaque index” OR “gingival index” OR “bleeding on probing” OR “probing depth” OR “tooth loss” OR “missing teeth” OR “marginal bone level” OR “IL-1b” OR “IL-8” OR “IL-6” OR “TNF-a” OR “MMP-1” OR “MMP-8” OR “IFN-y” OR “IL-4” OR “IL-9” OR “IL-10” OR “IL-13” OR “OPG” OR “RANK-L”) AND TITLE-ABS-KEY (cigarette OR “e-cigarette” OR “vaping cigarette” OR “electronic cigarette” OR “electronic nicotine delivery system” OR “Heat-Not-Burn Tobacco” OR vape OR vaping) AND PUBDATETXT (2022)
Cochrane Library From 2022 to 23 December 2025 English language	((“Periodontal disease” OR periodontitis OR “peri-implant disease” OR “peri-implantitis” OR “dental implant” OR “implant loss” OR “plaque index” OR “gingival index” OR “bleeding on probing” OR “probing depth” OR “tooth loss” OR “missing teeth” OR “marginal bone level” OR “IL-1b” OR “IL-8” OR “IL-6” OR “TNF-a” OR “MMP-1” OR “MMP-8” OR “INF-y” OR “IL-4” OR “IL9” OR “IL-10” OR “IL-13” OR “OPG” OR “RANK-L”)):ti,ab,kw AND ((cigarette OR “e-cigarette” OR “vaping cigarette” OR “electronic cigarette” OR “electronic nicotine delivery system” OR “Heat-Not-Burn Tobacco” OR vape OR vaping)):ti,ab,kw
