# Supplementary material for: Periodontal and Peri-Implant Status Among Users of Different Inhaled Tobacco and Nicotine Products: A Systematic Review
Source: Dent J (Basel). 2026 Sep 3;14(9):562. doi: 10.3390/dj14090562 (PMC13605147; doi:10.3390/dj14090562)
Supplement: Supplementary file 1 [file dentistry-14-00562-s001.zip › Table Risk of Bias.pdf]

SUPPLEMENTARY FILE S2 – RISK OF BIAS

Table S1. Risk of bias was assessed using the ROBINS-I tool to establish the overall quality of the included studies in the systematic review. Response to each question was defined for each item according to the ROBINS-I tool.

First Authors, Year Reference	Bias due to confounding (from question 1.1 to 1.8) Risk of bias judgment	Bias in the selection of participants (from question 2.1 to 2.5) Risk of bias judgment	Bias in measurement classification of interventions (from question 3.1 to 3.3) Risk of bias judgment	Bias due to deviations from intended interventions (from question 4.1 to 4.6) Risk of bias judgment	Bias due to missing data (from question 5.1 to 5.5) Risk of bias judgment	Bias in measurement of outcomes (from question 6.1 to 6.4) Risk of bias judgment	Bias due to the selection of the reported result (from question 7.1 to 7.3) Risk of bias judgment	Overall risk of bias judgment
Ahmed A.R., 2022 [18]	PY/N/NA/PN/PY/N/PY/PY Moderate	N/NA/NA/Y/NA	Y/Y/PN Low	N/NA/Y/Y/Y/NA Low	Y/N/N/NA/NA Low	N/N/Y/N Low	PN/PN/PN Low	Moderate
Al-Abdaly M.M. A.A., 2022 [46]	N/NA/NA/Y/Y/N/Y/NA Low	N/NA/NA/Y/NA Low	N/Y/PY Serious	PN/NA/Y/Y/Y/NA Low	Y/N/N/NA/NA Low	PN/Y/Y/PN Moderate	N/N/N Low	Serious
Ali D., 2022 [19]	N/NA/NA/Y/Y/N/Y/NA Low	N/NA/NA/Y/NA Low	Y/Y/Y/PN Low	N/NA/Y/Y/Y/NA Low	Y/N/N/NA/NA Low	N/N/Y/N Low	PN/N/N Low	Low
Ali D., 2022 [20]	N/NA/NA/Y/Y/N/Y/NA Low	N/NA/NA/Y/NA Low	Y/Y/Y/PN Low	N/NA/Y/Y/Y/NA Low	Y/N/N/NA/NA Low	N/N/Y/N Low	PN/N/N Low	Low
Ali D., 2023 [21]	N/NA/NA/Y/Y/N/Y/NA Low	N/NA/NA/Y/NA Low	Y/Y/Y/PN Low	PN/NA/Y/Y/Y/NA Low	Y/N/N/NA/NA Low	N/N/Y/N Low	PN/N/N Low	Moderate
Aljudabaidi S.M., 2025 [22]	PN/NA/NA/Y/Y/N/Y/NA Low	N/NA/NA/Y/NA Low	Y/Y/Y/N Low	N/NA/Y/Y/Y/NA Low	PN/N/N/N/PY Moderate	N/N/Y/N Low	N/N/N Low	Moderate
Alkan B., 2023 [23]	N/NA/NA/Y/Y/N/Y/NA Low	PY/N/N/Y/N Serious	Y/Y/N Low	N/NA/Y/Y/Y/NA Low	Y/N/N/NA/NA Low	N/N/Y/N Low	NI/Y/N Serious	Serious
Almazyad R.K., 2024 [24]	Y/N/N/PN/PN/PY/PN/NA Serious	N/NA/NA/PY/PN Moderate	PY/Y/PN Low	PN/NA/NI/PY/Y/NA Low	Y/N/N/NA/NA Low	PN/Y/Y/NI Serious	PN/PN/PN Low	Serious
Almeslet A.S., 2024 [25]	PN/NA/NA/PY/Y/Y/NA Low	N/NA/NA/N/PY Moderate	Y/Y/PN Low	N/NA/Y/Y/Y/NA Low	Y/N/N/NA/NA Low	N/N/Y/N Low	N/N/N Low	Low
Almeslet A.S., 2024 [26]	N/NA/NA/N/PY/Y/NA Low	N/NA/NA/PY/NA Low	Y/Y/PN Low	N/NA/Y/Y/Y/NA Low	Y/N/N/NA/NA Low	PN/PY/Y/N Moderate	PN/N/N Low	Moderate
Almeslet A.S., 2025 [27]	PY/N/NA/N//NA/N Critical	N/NA/NA/Y/NA Low	Y/Y/N Low	N/NA/Y/Y/Y/NA Low	Y/N/N/NA/NA Low	N/N/Y/N Low	N/N/PY Moderate	Critical
Alnufaiy B., 2025 [28]	N/NA/NA/Y/Y/N/Y/NA Low	N/NA/NA/Y/NA Low	Y/Y/Y/PN Low	N/NA/Y/Y/Y/NA Low	Y/N/N/NA/NA Low	N/N/Y/N Low	N/N/N Low	Low

Alqahtani A.S., 2022 [29]	N/NA/NA/Y/Y/N/Y/NA<br>Low	N/NA/NA/Y/NA<br>Low	Y/Y/PY/PN<br>Low	N/NA/Y/Y/Y/NA<br>Low	Y/N/N/NA/NA<br>Low	N/N/Y/N<br>Low	PN/PY/N<br>Moderate	Moderate
Alshahrani A., 2023 [30]	N/NA/NA/PY/Y/N/Y/NA<br>Low	N/NA/NA/Y/NA<br>Low	Y/Y/Y/N<br>Low	N/NA/Y/Y/Y/NA<br>Low	Y/N/N/NA/NA<br>Low	N/N//Y/N<br>Low	N/N/N<br>Low	Low
AlZamil A.F., 2023 [31]	N/NA/NA/Y/Y/N/Y/NA<br>Low	PY/PN/NA/Y/NA<br>Moderate	Y/Y/N<br>Low	N/NA/Y/Y/NA<br>Low	Y/N/N/NA/NA<br>Low	PN/Y/PN/N<br>Serious	PN/PY/PN<br>Moderate	Serious
Božac E., 2024 [32]	N/NA/NA/PY/Y/N/Y/NA<br>Low	PY/PY/Y/PY/NA<br>Serious	Y/Y/N<br>Low	N/NA/Y/Y/Y/NA<br>Low	Y/N/N/NA/NA<br>Low	PN/PY/Y/N<br>Moderate	N/N/N<br>Low	Serious
Dewan H., 2023 [33]	PN/NA/NA/PY/Y/Y/NA<br>Low	PN/NA/NA/PY/NA<br>Low	Y/Y/Y/PN<br>Low	N/NA/Y/Y/Y/NA<br>Low	Y/N/N/NA/NA<br>Low	PN/PY/Y/N<br>Moderate	PN/PY/PN<br>Moderate	Moderate
Gajendran P., 2022 [34]	N/NA/NA/PY/Y/N/Y/NA<br>Low	N/NA/NA/Y/NA<br>Low	Y/Y/Y/N<br>Low	N/NA/Y/Y/Y/NA<br>Low	Y/N/N/NA/NA<br>Low	PN/NI/Y/N<br>Low	N/N/N<br>Low	Low
Hasan N.W.M., 2024 [35]	N/NA/NA/Y/Y/N/Y/NA<br>Low	N/NA/NA/Y/NA<br>Low	Y/Y/Y/PN<br>Low	N/NA/Y/Y/Y/NA<br>Low	Y/N/N/NA/NA<br>Low	N/N/Y/N<br>Low	PN/N/N<br>Low	Low
Jabbar Najim R.A., 2025 [36]	N/NA/NA/Y/Y/N/Y/NA<br>Low	N/NA/NA/Y/NA<br>Low	Y/Y/Y/N<br>Low	N/NA/Y/Y/Y/NA<br>Low	Y/N/N/NA/NA<br>Low	N/N/Y/N<br>Low	PN/N/N<br>Low	Low
Li X, 2025 [37]	N/NA/NA/Y/qlY/N/Y/NA<br>Low	PN/NA/NA/NI/NA<br>Low	Y/Y/PN<br>Low	N/NA/Y/Y/Y/NA<br>Low	Y/N/N/NA/NA	PN/PY/Y/N<br>Moderate	Y/PY/PY<br>Critical	Critical
Mayer Y., 2025 [38]	N/NA/NA/Y/Y/N/Y/NA<br>Low	N/NA/NA/Y/NA<br>Low	Y/Y/Y/N<br>Low	N/NA/Y/Y/Y/NA<br>Low	Y/N/N/NA/NA<br>Low	N/N/Y/N<br>Low	N/N/N<br>Low	Low
Mišković I., 2024 [39]	N/NA/NA/Y/Y/N/Y/NA<br>Low	N/NA/NA/Y/NA<br>Low	Y/Y/N<br>Low	N/NAY/Y/Y/NA<br>Low	Y/N/N/NA/NA<br>Low	PN/NI/Y/N<br>Low	N/N/N<br>Low	Low
Oyapero A., 2023 [40]	Y/N/NA/Y/Y/PN/Y/NA<br>Moderate	PN/NA/NA/PY/NA<br>Low	Y/Y/N<br>Low	PN/NA/Y/Y/Y/NA<br>Low	PN/PY/N/NI/Y<br>Moderate	PN/PY/Y/N<br>Moderate	N/N/N<br>Low	Moderate
Saxena V., 2025 [41]	PY/N/N/N/PN/PY/PN/PN<br>Serious	PY/NA/NA/Y/NA<br>Low	PY/Y/PN<br>Low	PN/NA/Y/Y/Y/NA<br>Low	Y/N/N/NA/NA<br>Low	PY/PY/Y/N<br>Moderate	PY/Y/Y	Critical
Shah R., 2024 [47]	N/NA/NA/Y/Y/N/Y/NA<br>Low	N/NA/NA/Y/NA<br>Low	N/Y/N<br>Serious	N/NA/Y/Y/Y/NA<br>Low	Y/N/N/NA/NA<br>Low	PY/PY/Y/N<br>Moderate	N/N/N<br>Low	Serious
Sing D., 2024 [42]	Y/N/NA/N/N/PY/N/N<br>Critical	N/NA/NA/PY/NA<br>Low	N/Y/N<br>Serious	N/NA/Y/Y/Y/NA<br>Low	Y/N/N/NA/NA<br>Low	PN/PY/PY/NI<br>Serious	PY/Y/PY<br>Serious	Critical
Soldati K.R., 2022 [43]	N/NA/NA/Y/Y/N/Y/NA<br>Low	N/NA/NA/Y/NA<br>Low	PN/Y/N<br>Serious	N/NA/Y/Y/Y/NA<br>Low	Y/N/N/NA/NA<br>Low	PN/PY/Y/N<br>Moderate	PY/N/N<br>Serious	Serious
Suman A., 2025 [44]	N/NA/NA/Y/Y/N/Y/NA<br>Low	N/NA/NA/Y/NA<br>Low	Y/Y/N<br>Low	N/NA/Y/Y/Y/NA<br>Low	Y/N/N/NA/NA<br>Low	PN/PY/Y/N<br>Moderate	PN/N/N<br>Low	Moderate
Tanik A., 2022 [45]	N/NA/NA/PY/Y/Y/Y/NA<br>Low	PY/N/PN/Y/NA<br>Moderate	Y/Y/N<br>Low	N/NA/NI/NI/Y/NA<br>Low	PN/PY/N/NI/Y<br>Moderate	PN/PY/Y/N<br>Moderate	N/N/N<br>Low	Moderate
Taskaldiran E.S., 2024 [48]	N/NA/NA/Y/Y/N/Y/NA<br>Low	N/NA/NA/Y/NA<br>Low	Y/Y/N<br>Low	PN/NA/Y/Y/NA<br>Low	PY/PY/PY/Y/PY<br>Moderate	PN/PY//Y/N<br>Moderate	NI/NI/N<br>Low	Moderate

Abbreviations: yes “Y”; probably yes, “PY”; probably no, “PN”; no, “N”; no information, “NI”.
